# Glycerol-3-phosphate dehydrogenase (GPDH) gene family in *Zea mays* L.: Identification, subcellular localization, and transcriptional responses to abiotic stresses

**DOI:** 10.1371/journal.pone.0200357

**Published:** 2018-07-10

**Authors:** Ying Zhao, Xin Li, Feng Wang, Xunchao Zhao, Yuqiao Gao, Changjiang Zhao, Lin He, Zuotong Li, Jingyu Xu

**Affiliations:** 1 Key Lab of Modern Agricultural Cultivation and Crop Germplasm Improvement of Heilongjiang Province, Daqing Key Lab of Straw Reclamation Technology Research and Development, College of Agriculture, Heilongjiang Bayi Agricultural University, Daqing, People's Republic of China; 2 Heilongjiang Academy of Agricultural Sciences, Harbin, People's Republic of China; National Botanical Research Institute CSIR, INDIA

## Abstract

Glycerol-3-phosphate dehydrogenase (GPDH) catalyzes the formation of glycerol-3-phosphate, and plays an essential role in glycerolipid metabolism and in response to various stresses in different species. In this study, six *ZmGPDH* genes were obtained by a thorough search against maize genome, and designated as *ZmGPDH1-6*, respectively. The structural and evolutionary analyses showed that the ZmGPDHs family had typical conserved domains and similar protein structures as the known GPDHs from other plant species. ZmGPDHs were divided into NAD^+^-dependent type A form (ZmGPDH1-5) and FAD-dependent type B form (ZmGPDH6) based on their N-terminal sequences. Four full length ZmGPDHs were fused with GFP fusion proteins, and their subcellular localization was determined. ZmGPDH1 and ZmGPDH3 were located to the cytosol and mainly recruited to the surface of endoplasmic reticulum (ER), whereas ZmGPDH4 and ZmGPDH5 were located in the chloroplast. The transcriptional analysis of the *ZmGPDHs* in different maize tissues revealed relatively high level of transcripts accumulation of *ZmGPDHs* in roots and early stage developing seeds. Furthermore, we examined the transcriptional responses of the six *GPDH* genes in maize under various abiotic stresses, including salt, drought, alkali and cold, and significant induction of *ZmGPDH*s under osmotic stresses was observed. Together, this work will provide useful information for deciphering the roles of GPDHs in plant development and abiotic stress responses.

## Introduction

Glycerol-3-phosphate (G3P) has been shown to serve as an important intermediary metabolite connecting multiple metabolic pathways such as glycerolipid synthesis, glycolysis and gluconeogenes [1,2]. Enhanced level of G3P after feeding glycerol to developing seeds of rape results in an increase of carbon flux into triacylglycerol (TAG) synthesis, providing direct evidence that the rate of G3P provision may limit the biosynthesis of glycerolipid in seeds [3]. Additionally, studies have demonstrated that the metabolism of G3P in a number of plants played important roles in their adaption to adverse stresses, including pathogenic microbes, salinity, freezing and anaerobic stresses [4–6]. As an inducer of systemic acquired resistance (SAR), G3P enhanced the resistance of distal tissues against pathogen infection [6,7]. Besides, the production of intracellular glycerol allows *Dunaliella salina* and yeast to survive in hyperosmotic stress by maintaining a relatively low intracellular sodium concentration [8–10].

There are two primary metabolic pathways for the synthesis of G3P in higher plants. In the first pathway, G3P is generated directly from dihydroxyacetone phosphate (DHAP) via NAD^+^-dependent glycerol-3- phosphate dehydrogenase (GPDH, EC 1.1.1.8), catalyzing the reduction of NADH and DHAP to form NAD^+^ and G3P. In the second pathway, G3P is produced via glycerol kinase (GK, EC 2.7.1.30)-mediated phosphorylation of glycerol [11].

Genes encoding GPDH have been characterized and cloned from algae and a few plant species, and most of these genes are proved to be key factors in response to different stresses [12,13,7]. In the model plant *Arabidopsis*, there are five isoforms of GPDH associating with different subcellular organelles: two cytosolic NAD^+^-dependent forms, two plastidic NAD^+^- dependent forms, and one mitochondrial FAD-linked form (EC 1.1.99.5) [14–16,7]. The FAD-GPDH encoded by *AtGPDHm1*, along with a cytosolic GPDH encoded by *AtGPDHc1*, could form a mitochondrial G-3-P shuttle that served as a critical route to maintain the homeostasis of the NADH/NAD^+^ ratio under stresses [15]. Previous studies have showed that *AtGPDHm1* and *AtGPDHc1* are regulated by adverse stress conditions, such as salinity, dehydration and oxygen availability, and *AtGPDHm1* is also coupled to mitochondrial respiration [16]. Chanda et al. (2011) identified that the gene *SFD1*/*GLY1* encoding plastid-localized GPDH was involved in pathogen defense responses by affecting the total G3P pool in *Arabidopsis* [12]. Similarly, knocking down *TaGLY1* in wheat may inhibit G3P accumulation and compromise the resistance to stripe rust (*Puccinia striiformis* f. sp. *tritici*) [17]. The expression of mushroom *GPDH* gene is dramatically induced by salt, dehydration and cold conditions, and over-expression of *PsGPD* can improve salt tolerance of transgenic rice [18].

Plant GPDHs also play important roles on glycerol lipid metabolism [19,20].The *sfd*/*gly1* mutants show severe impairment in plastidal glycerolipids pathway of *Arabidopsis* without any effects on the synthesis of cytosolic TAG, suggesting that defection in plastidic GPDH has no impacts on glycerolipids assembly in the cytosol [12]. Overexpression of *SFD1/GLY1* in *Arabidopsis* also increases plastidic lipid contents in transgenic rice plants which is accompanied by a high photosynthetic assimilation rate [21]. Besides, two out of five GPDH enzymes in halophilic microalga (*Chlamydomonas reinhardtii)* have been shown to be necessary for nutrition deficiency-induced TAG synthesis [22,23].

Although *GPDH* genes have been cloned from *Arabidopsis* and some algae species, there is not much information available on GPDH gene family from other higher plants. In this study, a genome-wide identification of a family of 6 *GPDH* genes from maize genome was carried out. The bioinfomatic investigation of gene structure, evolutionary relationships, syntenic relations, protein three-dimensional (3D) structures was conducted. The subcellular localization of 4 ZmGPDHs was demonstrated. We also analyzed the transcripts accumulation of *ZmGPDHs* in different tissues and developmental stages by real-time quantitative RT-PCR. Furthermore, the transcriptional level of *ZmGPDHs* was measured under various abiotic stresses, including salt, alkali, drought and cold treatments. Taken together, the present work will contribute to better understand the functions of GPDHs in plant development and stress responses.

## Materials and methods

### Identification and sequence analysis of GPDH genes in maize

To identify all GPDH genes from maize, a systematic BLASTP search was performed against the maize genome sequencing project database (http://www.maizesequence.org) and NCBI database (http://www.ncbi.nlm.nih.gov) using the published *A*. *thaliana* GPDHs as queries. The protein sequences of putative maize GPDH family members with the highest identity (>90%) and lowest E-value (<10^−10^) were downloaded. The candidates were further screened *via* Phytozome database (https://phytozome.jgi.doe.gov/pz/portal.html) and SMART database (http://smart.emblheidelberg.de) to confirm the presence of a NAD^+^-binding domain (PF01210) or a GPDH-type DAO domain (PF01266), and the distribution of conserved domains were visualized by IBS software, version 2.0 [24].

Information about the genetic characteristics of *ZmGPDHs*, including chromosome locations, ORF lengths and protein length were collected from the maize genome sequencing project database. The molecular weight and isoelectric point were determined on the SIB bioinformatics resource portal (http://expasy.org/). Subcellular localization and transit peptides were predicted using CELLO 2.5 (http://cello.life.nctu.edu.tw/) and TargetP 1.1 server (http://www.cbs.dtu.dk/services/TargetP/) [25,26].

### Phylogenetic, gene structure and synteny analysis of maize GPDH family

The full-length amino acid sequences of GPDH proteins from *Z*.*mays* (*ZmGPDH*), *A*.*thaliana* (*AtGPDH*), *O*.*sativa* (*OsGPDH*), *G*.*max* (*GmGPDH*), and *S*.*bicolor* (*SbGPDH*) were used for constructing the phylogenetic tree through ClustalW alignment and the unrooted neighbor joining method with the bootstrap values performed on 1000 replicates using MEGA 5.0 [27]. The exon-intron structures of *GPDH* genes in *Z*.*mays* and *A*.*thaliana* were confirmed using Gene Structure Display Server (http://gsds.cbi.pku.edu.cn/index.php), which aligns the respective coding sequences with corresponding genomic sequences. The coding sequences and genomic sequences of *GPDH* genes in *Z*.*mays* and *A*.*thaliana* were downloaded from maize genome sequencing project database and NCBI database. The intron and exon boundary of some *ZmGPDH* genes was also checked by gene cloning and sequencing. The syntenic blocks among maize, rice, soybean, sorghum and *Arabidopsis GPDH* genes were obtained from the plant genome duplication database (http://chibba.agtec.uga.edu/duplication/) [28]. The gene ID and chromosomal localization of all the *GPDH* genes used are available in S1 Table.

### Protein structure analysis by homology modeling

The tertiary structure of ZmGPDHs was generated by homology modeling techniques using the SWISS-MODEL workspace (http://swissmodel.expasy.org) [29]. To predict the theoretical position of NAD-binding and FAD-binding with ZmGPDHs, the 3D structure of *Homo sapiens* GPD1 (PDB code: 1XOX) and *Escherichia coli* GlpD (PDB code: 2R46) were used as template respectively [30,31]. The predicted 3D models of ZmGPDHs were assayed via SWISS-MODEL server for model quality estimation. The 3D structural models of ZmGPDHs were presented using the Jmol software, version 12.0 [23].

### *ZmGPDHs-GFP* vector construction and protein subcellular localization determination

The gene-specific primers (S3 Table) were designed and used to isolate the putative *GPDH* genes from an inbred maize variety He-344 (provided by the Maize Breeding Research Center of Heilongjiang Bayi Agricultural University, Daqing, China). The full-length coding regions of *ZmGPDH1*, *3*, *4*&*5* were amplified from He-344 using high-fidelity KOD-mediated (TOYOBO, Japan) RT-PCR, which were fused to the pBI121 vector containing green fluorescent protein (GFP) tags under control of the CaMV35S promoter. For co-localization studies, a far-red fluorescent protein mkate was used as the cytosol marker [32]. Besides, an endoplasmic reticulum (ER) marker PIN5 was fused with the far red fluorescent protein (mkate), generating an N-terminal mkate::PIN5 fusion driven by the CaMV35S promoter [33]. The rice protoplasts were isolated from the leaves of 7 days-old seedlings that was grown in a greenhouse under a weak light condition to minimize auto fluorescence of chlorophyll. The subcellular localization of the GFP expression in the rice mesophyll protoplasts was monitored by confocal microscopy 18 h after PEG-calcium mediated transformation as described by Yoo et al. [34].

### Plant materials and stress treatments

To analyze the tissue-specific transcriptional profiles of the *ZmGPDH* genes, maize variety He-344 was planted in the experimental field, and leaf, stem, root, sheath, anther, tassel, silk, pollen and subtending leaf samples were harvested at 65 days after planting. Samples of developing seeds were collected at 5, 10, 15, 20, 30 and 40 days after flowering (DAF). For the transcriptional analysis of *ZmGPDHs* under abiotic stress treatments, seedlings at three-leaf stage were treated with 200 mM NaCl, 150 mM NaHCO_3_ and 20%-PEG (molecular weight 6,000) solutions. Low temperature treatment was conducted by placing seedlings in 4°C incubator. Leaf samples were collected at 0, 1, 3, 6, 12 and 24 h after treatment, and samples from plants without treatment were used as controls.

### qRT-PCR analysis of *ZmGPDHs*

Total RNA was extracted using the Trizol reagent (Invitrogen, Carlsbad, CA, USA). One micrograms of each RNA sample was used as the template for first-strand cDNA synthesis using a ReverTra Ace qPCR RT Master Mix including gDNA Remover (TOYOBO, Japan). Real-time quantification PCR (qRT-PCR) was performed in an optical 96-well plate using a SYBR Select Master Mix RT-PCR System. *ZmGAPDH* and *ZmACTIN* were used for standardization of target genes [35,36]. Three independent biological replicates were carried out and the results of qRT-PCR were analyzed using the 2^-^ΔΔ^ct^method. All the primers used were shown in S3 Table.

### Statistical analysis

Data are presented as Mean±SD. The Student’s *t*-test was used to determine the significance levels using SPSS 21.0 software throughout this study. A P-value of <0.05 was considered statistically significant.

## Results

### Identification and isolation of GPDH gene family in maize

A total of six genes encoding glycerol-3-phosphate dehydrogenase (GPDH) enzymes were retrieved *via* BLAST searche against the maize genome using the reported *A*. *thaliana* GPDHs as queries, and they were designated as *ZmGPDH1-6*, respectively (Table 1). As shown in Table 1, the ORF of *ZmGPDH1-6* ranged from 1104 to 1890 bp, and the encoded proteins comprised 367–629 amino acid residues. The predicted molecular weight and isoelectric points of all ZmGPDH proteins ranged from 40.3kDa/5.48 to 68.2kDa/9.97, respectively.

**Table 1:** Characteristics of the maize GPDH genes (*ZmGPDHs*).

Gene Name	Gene ID^a^	Gene location	ORF^b^ length (bp)	Protein length	Isoelectric point	Molecular weight (KDa)	Subcellular localization
*ZmGPDH1*	GRMZM2G155348 _T01	ZM3: 152532919–152538365	1377	458	7.29	51.0	Cytoplasmic
*ZmGPDH2*	GRMZM2G090747 _T01	ZM8: 171085827–171089416	1395	464	6.79	51.4	Cytoplasmic
*ZmGPDH3*	GRMZM2G173195 _T01	ZM8: 120280265–120283927	1389	462	8.43	51.0	Cytoplasmic
*ZmGPDH4*	GRMZM6G161711 _T01	ZM3: 144431555–144433915	1104	367	5.48	40.3	Chloroplast
*ZmGPDH5*	GRMZM2G063258 _T05	ZM7: 21283001–21287121	1284	427	9.97	45.7	Chloroplast
*ZmGPDH6*	GRMZM2G446108 -T01	ZM10: 96895168–96924949	1890	629	8.28	68.2	Mitochondrial

^a^IDs are available in the maize genome sequencing project database (http://www.maizesequence.org).

^b^ORF: open reading frame.

**Fig 1:**
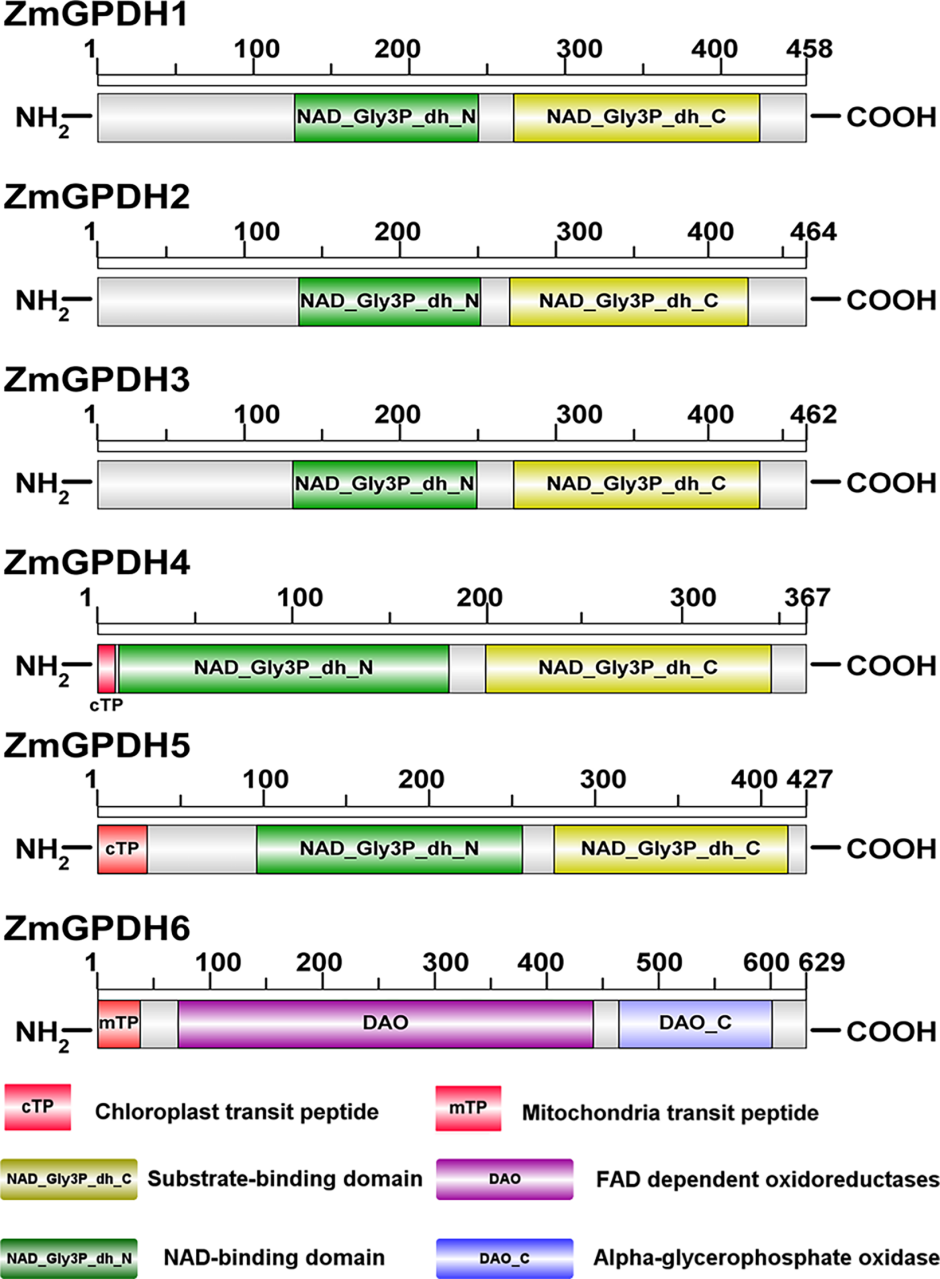
Diagram of the maize GPDH proteins showing the bi-domain structure. Predicted signal peptides are shown as colored rectangles. The numbered bar indicates the amino acid.

As shown in Fig 1, all of the six ZmGPDH proteins had a bi-domain protein structure. Specifically, *ZmGPDH1-5* encoded the NAD^+^-dependent GPDHs, containing a N-terminal NAD^+^-binding domain (PF01210) and a C-terminal NAD^+^-dependent GPD domain (PF07479) which represents the C-terminal substrate-binding site. *ZmGPDH6* encoded a FAD-dependent GPDH harboring a N-terminal FAD-dependent oxidoreductases domain (DAO, PF01266) and a C-terminal alpha-glycerophosphate oxidase domain (DAO_C, PF16901). Moreover, based on the prediction of subcellular localization, ZmGPDHs were divided into three types: three cytosolic NAD^+^-GPDHs (ZmGPDH1, 2&3), two plastidic NAD^+^-GPDHs (ZmGPDH4&5), and one mitochondrial FAD-GPDH (ZmGPDH6). The presentation of a predicted N-terminal chloroplast transit peptide in ZmGPDH4&5 and an apparent mitochondrial targeting sequence in ZmGPDH6 was in line with the *in silico* prediction (Fig 1).

### Phylogenetic analyses of ZmGPDH family

**Fig 2:**
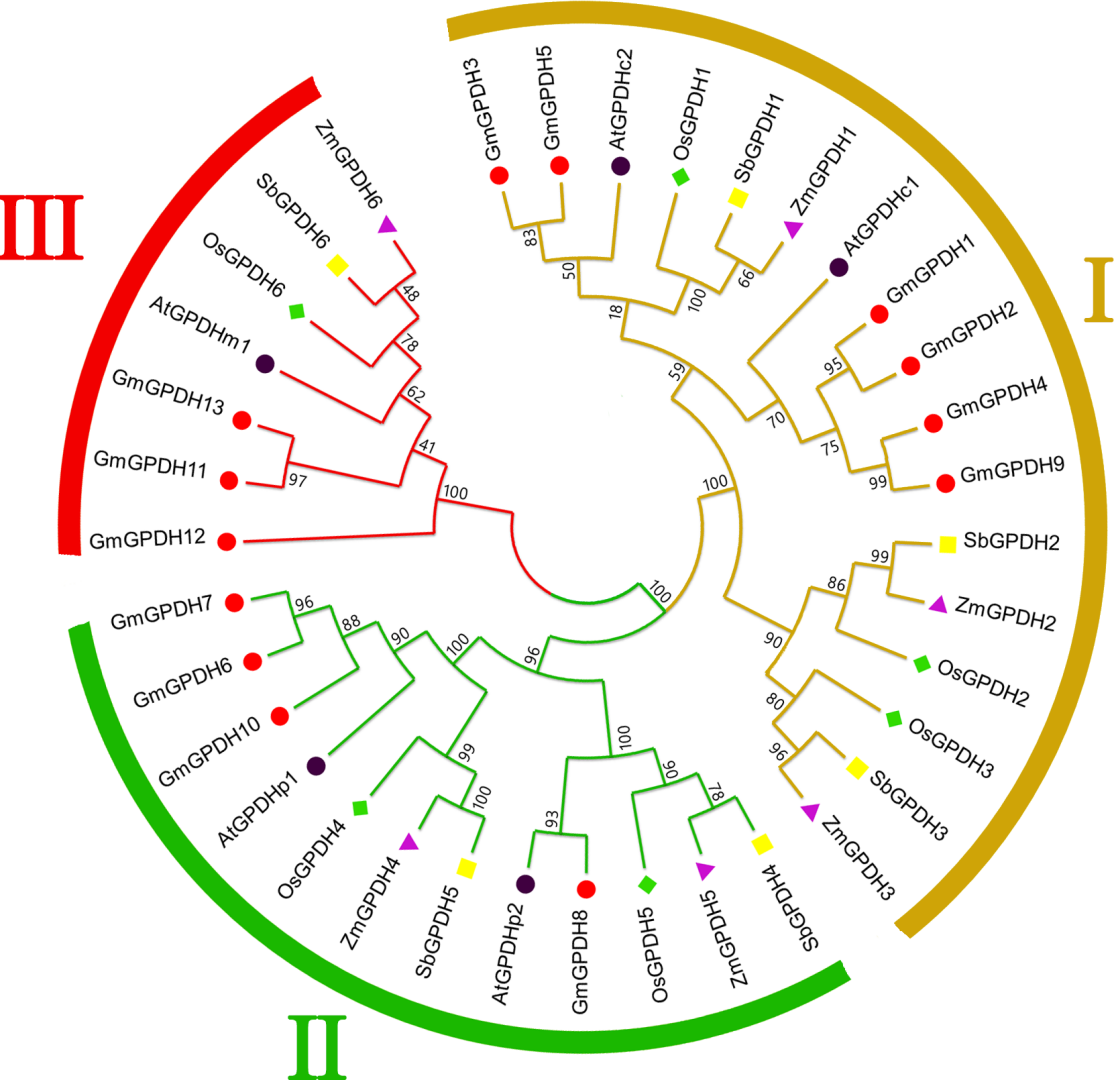
Phylogenetic tree of GPDH proteins from maize (purple triangles), rice (green diamonds), soybean (red circles), sorghum (yellow squares) and *Arabidopsis* (black circles). The full-length amino acid sequences of the GPDH proteins were used to construct the phylogenetic tree using the MEGA 5.0. The information of these genes can be seen in S1 Table.

To determine the evolutionary and phylogenic relationships of ZmGPDHs with homologous genes from other plant species, the full-length protein sequences of GPDHs s from *Arabidopsis* (*A*.*thaliana*, AtGPDH1-5), rice (*O*.*sativa*, OsGPDH1-6), soybean (*G*.*max*, GmGPDH1-13), and sorghum (*S*.*bicolor*, SbGPDH1-6) were aligned with ZmGPDH1-6, and a Phylogenetic tree was constructed (see S1 Table for the information of these genes). The Phylogenetic tree revealed that the plant GPDHs could be classified into three groups, as shown in Fig 2, corresponding to the GPDH cluster I, II, and III. ZmGPDH1,2&3 isoforms fell into cluster I, which encompassed two NAD^+^ -dependent cytosolic AtGPDHc isoforms, and orthologous genes from other species. ZmGPDH4&5 was subdivided into cluster II, along with two plastidic NAD^+^-dependent AtGPDHp isoforms. The cluster III contained ZmGPDH6, the mitochondrial FAD-dependent AtGPDHm and a small number of orthologous genes. Within each GPDH cluster, particular subgroups of paralogous and orthologous genes were identified, implying ancestral speciation or duplication events. For example, the AtGPDH isoforms were more closely related to the putative GPDH orthologs from soybean, which all belonged to the dicot family. However, the ZmGPDH isoforms shared high homology with their putative GPDH orthologs from monocot sorghum and rice, reflecting consistency in the evolution of GPDH isoforms and plant lineages. The specific localization feature of three clusters as symbolized by *Arabidopsis* GPDH isoforms suggested the relatedness of distinct function of each group of GPDH genes with their evolutionary process.

### Syntenic relations and exon-intron organization of *ZmGPDH* genes

**Fig 3:**
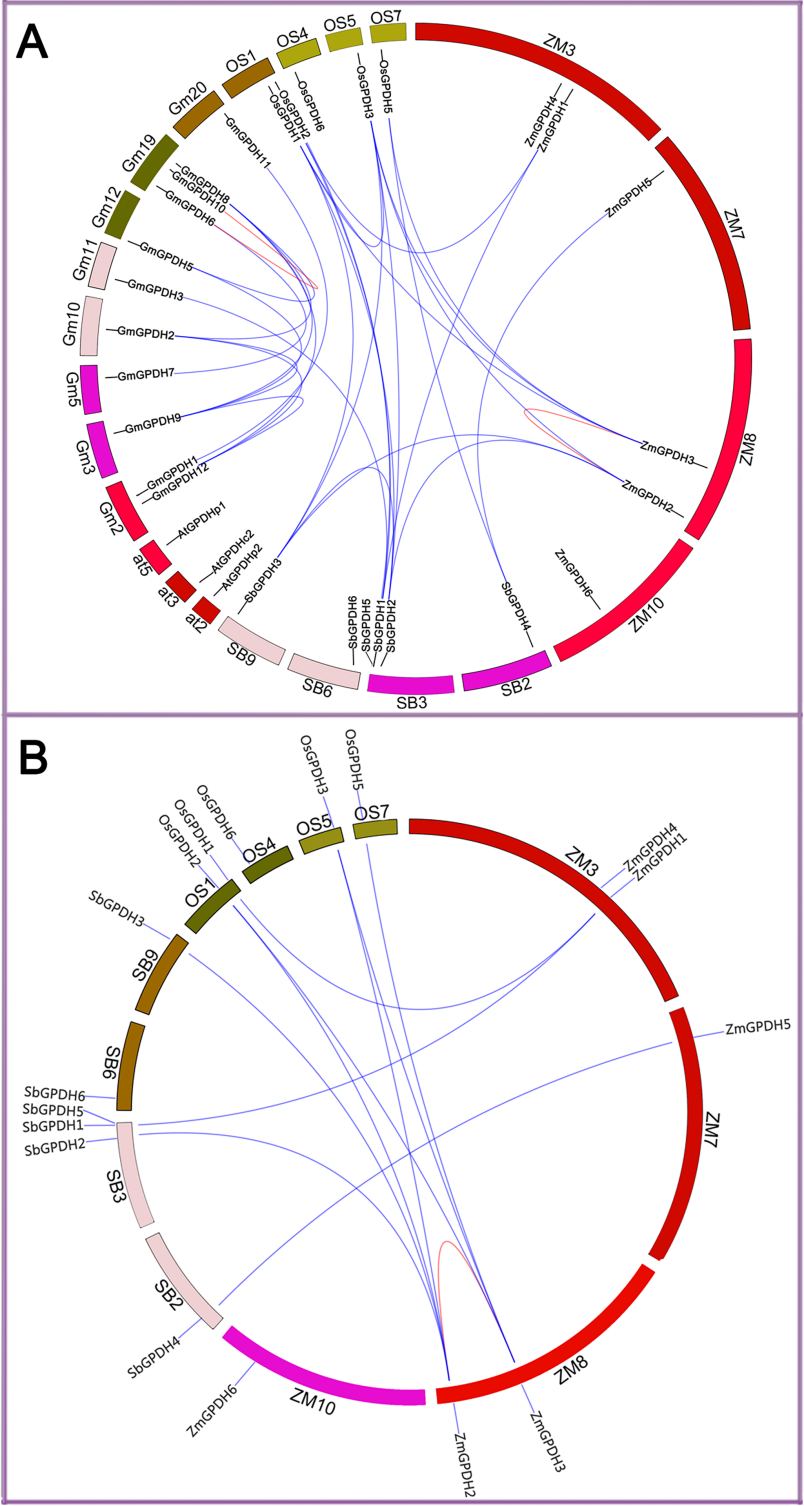
Syntenic analysis of *GPDH* genes from different plant species. (A) Syntenic analysis of maize, rice, soybean, sorghum and *Arabidopsis GPDH* genes. (B) Syntenic analysis of maize, rice and sorghum *GPDH* genes. The chromosomes are depicted as a circle. The colored curves denote the syntenic regions of the GPDH genes.

A syntenic analysis among *GPDH* genes from maize, *Arabidopsis*, soybean, rice and sorghum was performed, and their physical locations on chromosomes were shown in Fig 3. Six *ZmGPDH* genes were distributed on four out of ten maize chromosomes and each of the four chromosomes contained one to two *ZmGPDH* genes. In addition, through gene syntenic analysis, we identified 18 orthologous pairs of *GPDH* genes among maize, soybean, rice, sorghum, and *Arabidopsis*, and the orthologous gene pairs were apt to be found among homologous species (Fig 3A, S2 Table). The *GPDH* genes from maize had only one syntenic relationship with the *GPDH* genes from rice or sorghum that all belong to the monocot genera, including six orthologous gene pairs between maize and rice, and four orthologous gene pairs between maize and sorghum (Fig 3B). This result suggested that the *ZmGPDHs*, *OsGPDHs* and *SbGPDHs* might derive from the same ancestral genes, which was consistent with their evolutionary relationships. Additionally, one paralogous *GPDH* gene pair was found in each of the examined monocot species, while eleven paralogous gene pairs were identified in dicot soybean.

**Fig 4:**
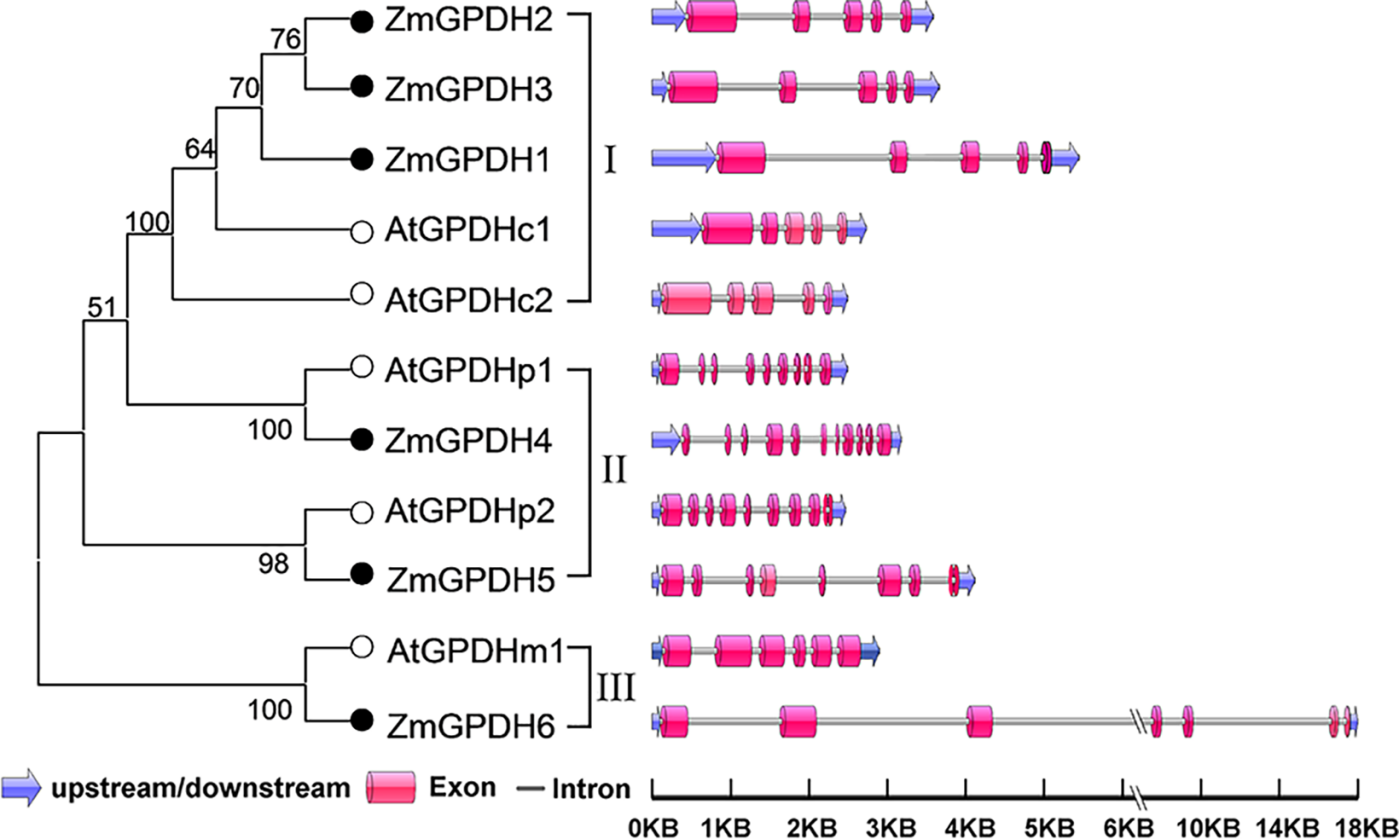
Structural analysis of maize and *Arabidopsis GPDH* genes. The phylogenetic tree of GPDH proteins from maize and *Arabidopsis* are shown on the left and are classified into three groups. The exon-intron organization is shown on the right, with exons and introns represented by pink boxs and gray lines, respectively, and untranslated regions indicated by blue arrow.

The gene structural analysis of maize and *Arabidopsis* GPDH were performed using the GSDS software. As shown in Fig 4, the exon-intron organization of *GPDH* genes was highly conserved within each phylogenic group. *GPDH* genes in group I exhibited a small and equal number of exons (5) and nearly identical exon lengths. *GPDH* genes in group II showed 90% sequence identity and had the similar exon-intron structure between maize and *Arabidopsis*, and the number of exons was among 8–11. *ZmGPDH6* and *AtGPDHm1* in group III contained seven exons and six exons respectively, and *ZmGPDH6* had a large intron lengh, which was different from other GPDHs. On the other hand, the full-length sequences of four *ZmGPDH* genes (*ZmGPDH1*, *3*, *4*&*5*) were successfully isolated and were verified by sequencing. The sequence alignment was carried out to validate the gene structure, and we found the intron-exon distribution and the intron-exon boundary was consistent with the *in silico* prediction (S2 File).The complete gene and cDNA sequences of these genes were deposited in GenBank with the following accession numbers: *ZmGPDH1* (MH483980, MH460963), Z*mGPDH*3 (MH483981, MH460964), *ZmGPDH4* (MH483982, MH460965), and *ZmGPDH*5 (MH483983, MH460966).

### 3D structure construction of ZmGPDH proteins

**Fig 5:**
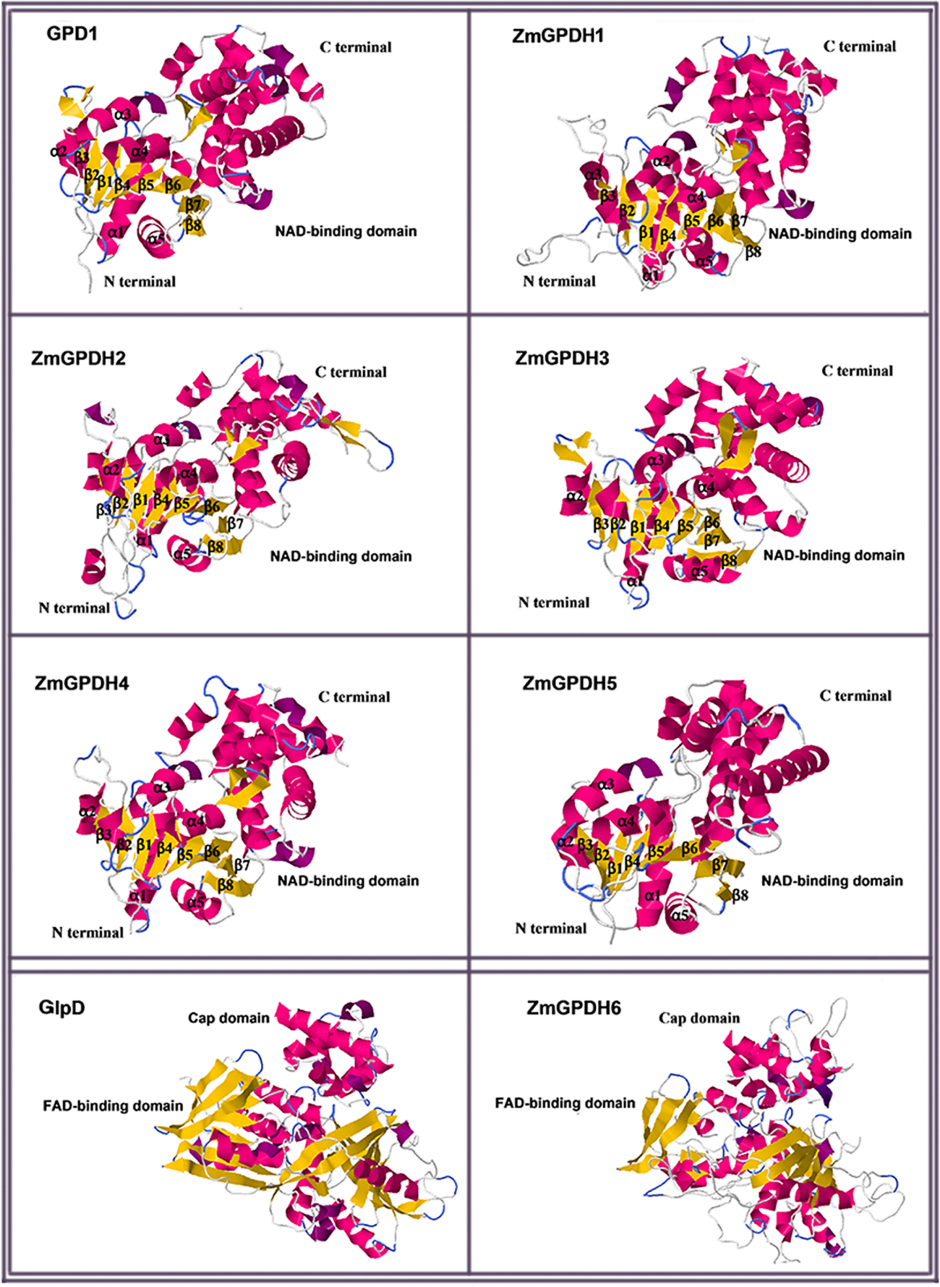
The cartoon representation of the predicted 3-dimensional structural models of ZmGPDH1-6. The 3D structure was generated by homology modeling at the SWISS-MODEL workspace, and the model of *Homo sapiens* GPD1 (PDB code: 1XOX) and *Escherichia coli* GlpD (PDB code: 2R46) were used as templates. The a-helices were colored in shocking pink and the β-sheet was shown by yellow arrow.

The three-dimensional structure of ZmGPDH proteins was predicted by homology modeling method. The prediction of ZmGPDH1-5 was based on the *Homo sapiens* GPD1 (PDB code: 1XOX) tertiary structure, which shared 43.06%, 37.25%, 42.86%, 50.87%, and 30.46% sequence identity with ZmGPDH1-5, respectively. The prediction of ZmGPDH6 was based on *Escherichia coli* GlpD (PDB code: 2R46) tertiary structure, which shared 34.18% sequence identity with ZmGPDH6. The quality assessment of 3D protein structures were performed *via* SWISS-MODEL server. The GMQE scores (the estimated model reliability between 0 and 1) of the predicted 3D models for ZmGPDH1-6 were 0.47, 0.47, 0.47, 0.77, 0.54 and 0.53, respectively. These data suggested that all the sequences of ZmGPDH1-6 matched the homologous templates well on the server, indicating that these protein models were reliable. ZmGPDH1-5 had very similar protein structure models. Similar to HsGPD1, the N-terminal NAD^+^-binding domain of ZmGPDH1-5 exhibited a stable “sandwich” shaped structure and comprised two major parts: a spatial symmetric β-sheet core and several helices (a1–a5) wrapping on both sides of the β-sheet core. The β-sheet core also contained a six-stranded parallel β-sheet (β1–β6) and two long anti-parallel strand β-sheets (β7, β8), which has been proved as the binding site of co-enzyme, such as NADH. On the other hand, the substrate-binding domain at the C-terminal was mainly composed of helices (Fig 5). Compared to ZmGPDH1-5, ZmGPDH6 had different crystal structure, which consisted of a soluble extra- membraneous “cap” domain at the C-terminal and a FAD-binding domain at the N-terminal (Fig 5).

### Subcellular localization of ZmGPDHs

**Fig 6:**
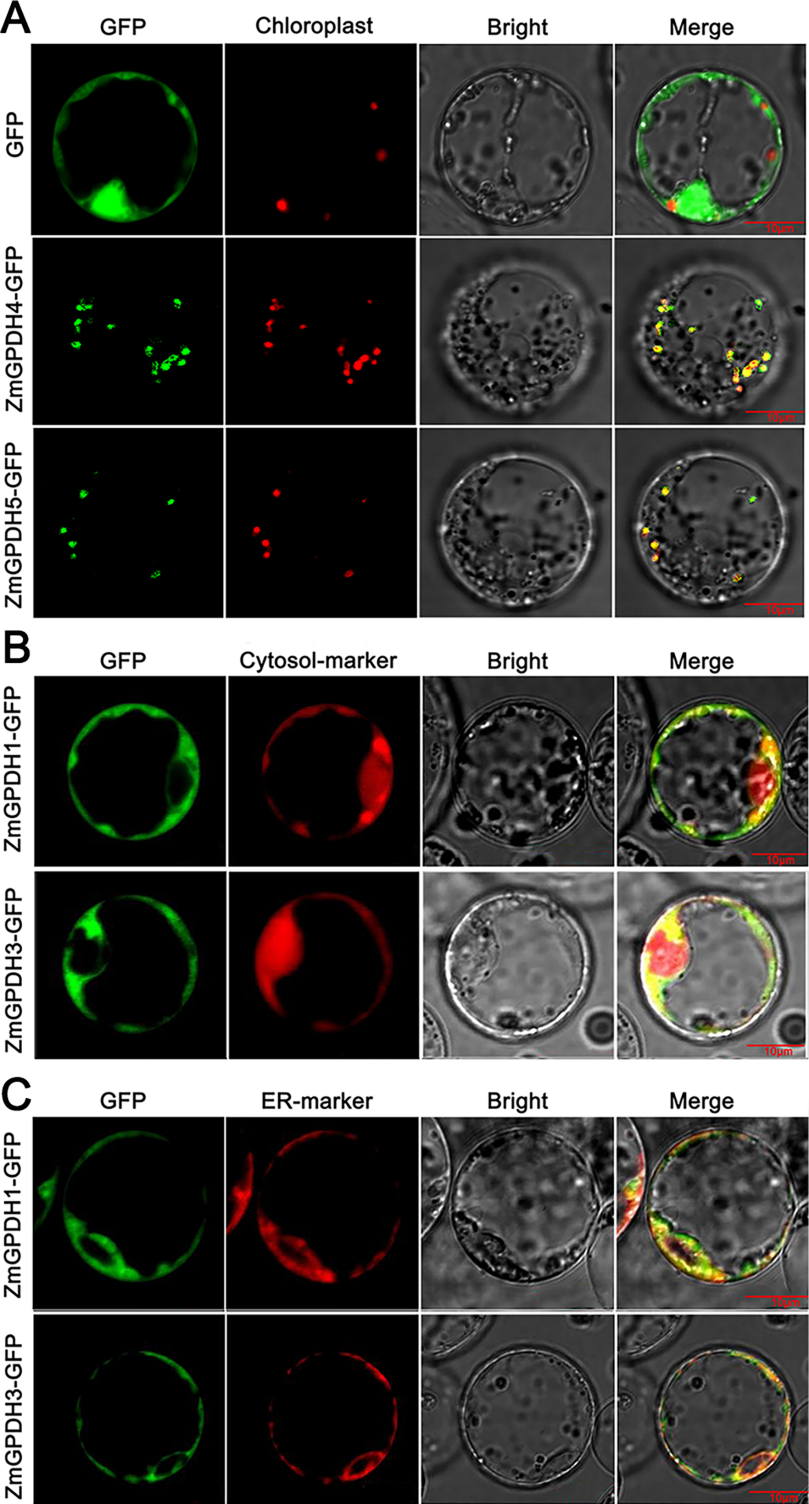
Subcellular localization of ZmGPDH-GFP fusion proteins. (A) Confocal micrographs showing localization of GFP, ZmGPDH4-GFP and ZmGPDH5-GFP. The merged pictures include the green fluorescence channel (first panels) and the chloroplast autofluorescence channel (second panels). (B) Confocal micrographs showing localization of ZmGPDH1-GFP and ZmGPDH3-GFP in mesophyll protoplasts validated a cytosol marker mkate (red). (C) Confocal micrographs showing localization of ZmGPDH1-GFP and ZmGPDH3-GFP in mesophyll protoplasts validated by a mkate-tagged ER marker PIN5 (red). The merged pictures of B and C include the green fluorescence channel (first panels) and the red fluorescence channel (second panels).

The subcellular localization of GPDHs is important for their functions. To look into the cellular localization of ZmGPDHs, the coding regions of four *ZmGPDH* genes (*ZmGPDH1*, *3*, *4*&*5*) were cloned and fused in-frame with green fluorescent protein (GFP) tags under control of the CaMV35S promoter. The GFP-tagged GPDH proteins and the control empty vectors containing GFP tags were temporarily expressed in rice mesophyll protoplasts, respectively, and visualized by a confocal laser-scanning microscope. As shown in Fig 6A, the control GFP was uniformly dispersed throughout the whole mesophyll protoplast except vacuole and chloroplast, whereas the ZmGPDH4 and ZmGPDH5 fusion proteins were exclusively located to the chloroplast of mesophyll cells, which was consistent with the *in silico *prediction. Nevertheless, The ZmGFPH1-GFP or ZmGFPH3-GFP fusion proteins were observed around the cytosol periphery, and also obviously associated with a reticular compartment circulating the nucleus-possibly endoplasmatic reticulum (ER) (S1 Fig). The co-expression of the cytosol-marker (mkate) or ER-marker (PIN5) together with ZmGPDH1 or ZmGPDH3 was examined in rice mesophyll protoplasts. The merged image of ZmGPDH-expressed rice cells stained with a cytosol marker confirmed that the ZmGPDH1-GFP or ZmGPDH3-GFP fusion proteins were specifically distributed in cytosol, while the marker proteins were dispersed over cytosol as well as nucleus (Fig 6B). In addition, ZmGPDH1 or ZmGPDH3 proteins exhibited similar subcellular distributions with ER-marker which also showed their putative ER localization (Fig 6C). Together, these results speculated that ZmGPDH1-GFP and ZmGPDH3-GFP fusion proteins were located in cytosol and closely adjacent to ER, which was reasonable since the biosynthesis of glycerolipids mainly occurred in ER.

### Transcript accumulation of *ZmGPDHs* in different tissues and developmental stages

**Fig 7:**
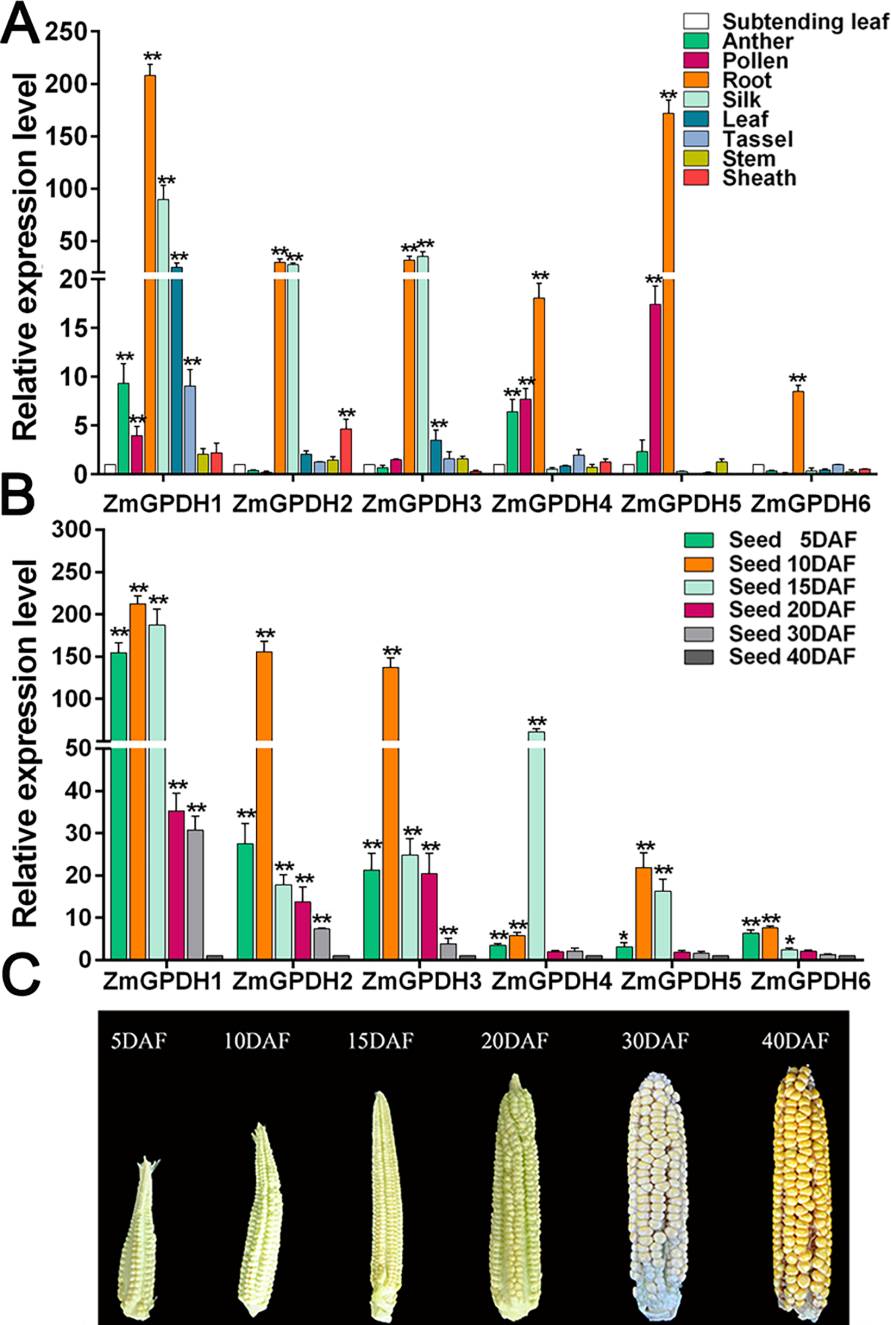
The differential transcript profiling of *ZmGPDH* genes in different tissues and developmental stages. (A) The differential transcript accumulation of *ZmGPDH* genes in maize tissues. The transcripts of *ZmGPDHs* in subtending leaf was used as a calibrator. (B) The differential transcripts accumulation of *ZmGPDHs* in developing seeds at 5, 10, 15, 20, 30 and 40 days after flowering (DAF). The transcripts of *ZmGPDHs* in developing seeds at 40DAF was used as a calibrator. (C) The physiological phenotype in developing seeds at 5, 10, 15, 20, 30 and 40 days after flowering (DAF). The asterisks indicate that the corresponding genes are significantly up or down-regulated in different tissues, as determined by the Student’s *t*-test (*P < 0.05, ** P < 0.01).

To investigate the functions of *GPDH* genes in maize development, the transcripts of *ZmGPDHs* were analyzed in different tissues (leaf, stem, root, sheath, anther, tassel, silk, pollen and subtending leaf), and multiple stages of developing seed (5, 10, 15, 20, 30 and 40 days after flowering) by quantitive real-time RT-PCR. As shown in Fig 7A, the *ZmGPDHs* exhibited tissue specific transcript profiles. The transcripts of *ZmGPDH1* could be detected in all examined tissues, with a high transcriptional level in silk, root and leaf; the transcripts of *ZmGPDH2* was mainly detected in silk and sheath, and *ZmGPDH3* was specifically expressed in silk and leaf. The transcripts of *ZmGPDH4* and *ZmGPDH5* were mainly shown in pollen and anther. In addition, the transcriptional level of *ZmGPDH6* was low in all tested tissues except root. The tissue-specific transcript profiling suggested that *ZmGPDHs* might play versatile biological roles in maize growth and development.

The transcript accumulations of *ZmGPDH* genes were examined in developing seeds at multiple stages. As demonstrated in Fig 7B, all *ZmGPDH* genes maintained comparatively high transcriptional levels at the early seed development stages (5, 10 and 15 DAF) and a low level of transcription at the late seed developmental stages (20, 30 and 40 DAF). Most of the *ZmGPDH* genes showed the highest level of transcripts in developing seeds at 10 DAF, whereas the transcripts of *ZmGPDH4* were peaked in seeds at 15 DAF. Compared with other *ZmGPDH* genes, the transcript accumulation of *ZmGPDH1 *was the highest at all seed developing stages, whereas the transcriptional levels of *ZmGPDH6* were relatively low throughout the tested stages.

### Transcript accumulation of *ZmGPDHs* in response to abiotic stress treatment

**Fig 8:**
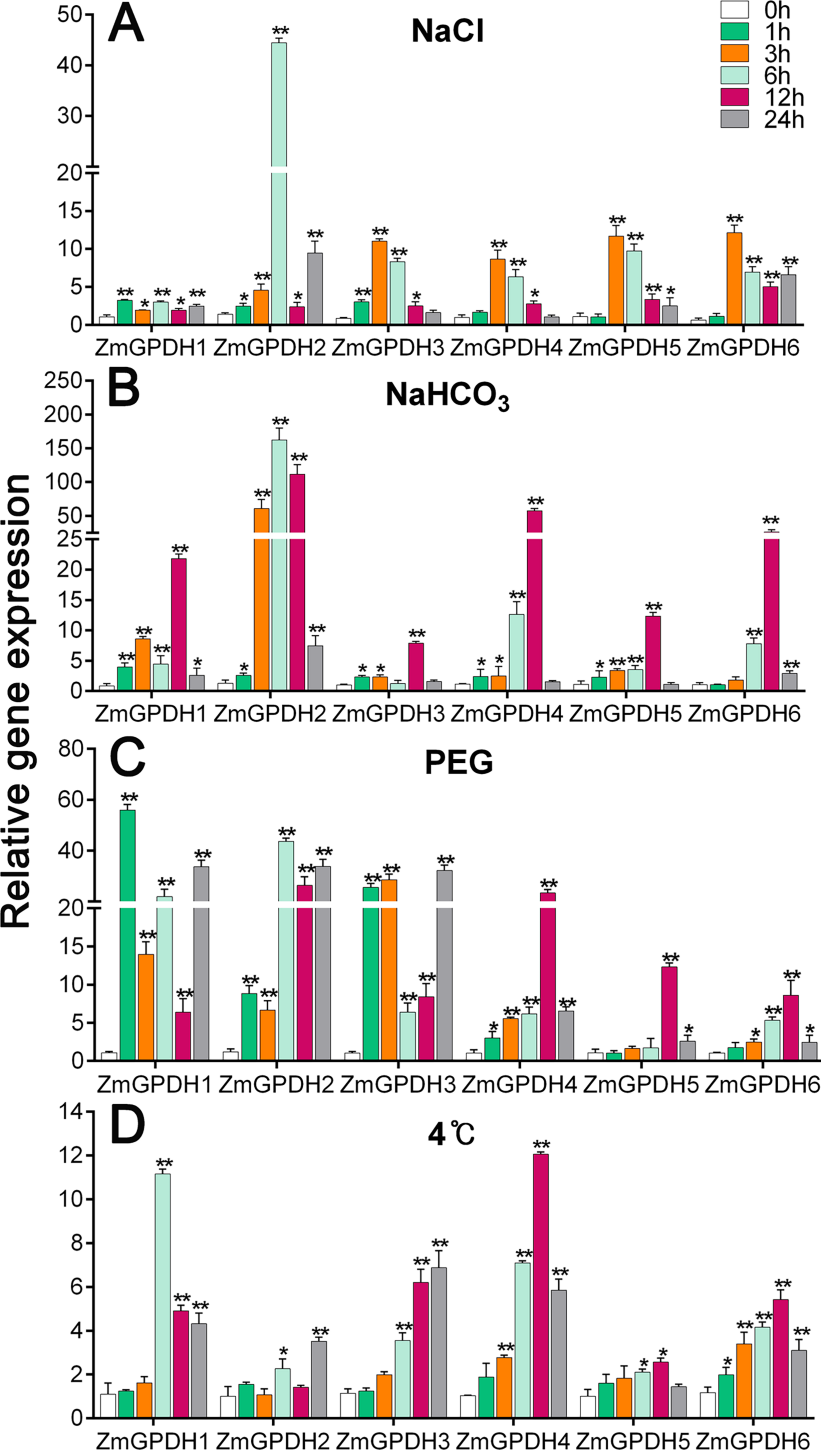
The transcript profiling of *ZmGPDHs* in response to NaCl (A), NaHCO_3_ (B), PEG (C), 4°C (D) treatments in leaves of maize seedlings. The transcripts of *ZmGPDHs* in control environment was used as a calibrator. The asterisks indicate that the corresponding genes were significantly up or down-regulated in response to different treatments, as determined by the Student’s *t*-test (*P < 0.05, ** P < 0.01).

*GPDH* genes have been proved to be essential in mediating stress responses in *yeast*, marine algae and *Arabidopsis* [9,4,7]. The analysis of promoter sequences of *ZmGPDHs* revealed a number of *cis*-elements, including LTR, TC-rich repeats, HSEs and MBS (S2 Fig), which are likely related to salt, drought and low temperature stresses maize frequently encountered in our area (northeast China). Therefore, to further understand how *ZmGPDH* genes respond to abiotic stresses, their transcript accumulations were analyzed in three-leaf stage maize seedlings exposed to various stress conditions, including salt (200 mM NaCl), alkali (150 mM NaHCO_3_), drought (20% PEG) and low temperature (4°C). As shown in Fig 8A, all of the *ZmGPDHs* were apparently up-regulated at the early stage of salt treatment. *ZmGPDH1*,*2*&*3* were initially induced after 1 h, maintaining relatively high transcripts level during the treatment, and the *ZmGPDH4*,*5*&*6* were remarkably up-regulated after 3 h. In response to alkaline treatment, most *ZmGPDHs* were significantly up-regulated, especially *ZmGPDH2*, which had notably higher level of transcriptsat 6 h (Fig 8B). In addition, all of the *ZmGPDH* genes exhibited the highest transcripts level at 6h or 12h of alkaline treatment, and the transcriptional levels of *ZmGPDH2* during the middle or late stage of treatment were much higher than other genes.

There were large differences on transcription of *ZmGPDHs* under drought treatment (Fig 8C). Of the six *ZmGPDH* genes, *ZmGPDH1* was the most drought inducible, reaching its maximum transcriptional level after 1 h treatment. *ZmGPDH2* and *ZmGPDH3* showed the highest transcriptional level at 6h and 24h respectively, while *ZmGPDH4*,*5*&*6* exhibited the highest transcriptional level at 12h. Notably, the transcripts of *ZmGPDH5*&*6* were much lower than that of others during the drought treatment except the 12h time point. Compared to other treatments, the overall transcripts of *ZmGPDHs* under the cold treatment were relatively low (Fig 8D). The *ZmGPDH1* transcripts were dramatically up-regulated at 6h under low temperature treatment, and the transcripts of *ZmGPDH2*&*3* were initially up-regulated after 3h, and peaked at 24h. In contrast, *ZmGPDH4*,*5*&*6* represented the maximum transcripts at 12h under cold treatment.

## Discussion

Maize is a major cereal crop in the world as well as an important source for animal feed, corn oil, industrial materials, and biofuels [37]. An increasing amount of research has indicated that *GPDH* genes play significant roles during plant growth and in response to various stresses [38,17,7,21]. Although *GPDH* genes have been cloned from *Arabidopsis* and some algae species, there is not much information available on GPDHs from other higher plants. In the present work, we identified a *GPDH* gene family encoding six GPDHs in maize. Based on the prediction of subcellular localization and the sequences of N-terminal, ZmGPDHs were divided into three types: three cytosolic NAD^+^-GPDHs (ZmGPDH1, 2&3), two plastidic NAD^+^-GPDHs (ZmGPDH 4&5), and one mitochondrial FAD-GPDH (ZmGPDH6). Phylogenic analysis resulted in the same classification of ZmGPDHs by subdividing them into three clusters, which suggested the relatedness of distinct function of each group of *GPDH* genes with their evolutionary process.

All ZmGPDH proteins contain the specific and/or necessary protein domains (NAD_-_Gly3P_-_ dh_-_ N, NAD_-_Gly3P_-_dh_-_C, DAO, DAO_C), which may be significant for functional conservation. It has been reported that some *GPDH* genes from the genera *Dunaliella* and *Chlamydomonas* also have a phosphoserine phosphatase (PSP) domain which functions as glycerol-3 -phosphatase and catalyzes the step transfering dihydroxyacetone phosphate (DHAP) to glycerol directly [23,39,40]. Nevertheless, we did not found any PSP domain in ZmGPDH proteins. Moreover, the previously reported NAD^+^-dependent GPDs exhibited an identical binding frame analogous to GXGXXG, in which the first two glycines take part in NAD^+^-binding, and the third promotes close packing of the helix to the beta-strand [14]. Similarly, we found that ZmGPDH1 and ZmGPDH2 contained the conserved GAGAWG motif at residues 44–50, and ZmGPDH4 had a GSGNWG motif at residues 16–21. Interestedly, ZmGPDH3 had a GARAWG motif at residues 42–48 similar to GAGAWG, while the effect of this G to R variation needed to be further studied (S3 Fig). The FAD-dependent ZmGPDH6 shared no sequence homology with other NAD^+^-GPDHs in maize, while a putative FAD-binding motif (DVLVIGGGATGCGVALDAVTRGLRVGLVER) as well as sequences involved in G-3-P binding (DVLSAWSGIRPLA; GLITITGGKWTTYRSMAE) were identified (S3 Fig).

Additionally, the phylogenic, gene structural and syntenic analysis provided functional and evolutionary connections among *GPDH* genes from different plant species. We found that all *ZmGPDH* genes showed close phylogenetic relationships with the putative sorghum *GPDH* genes, which is consistent with their evolutionary relationship, implying possible functional conservation (Fig 2). Furthermore, four Zm*GPDH* genes were found having collinear relationships with monocot rice and sorghum *GPDH* genes, suggesting that most *ZmGPDH* genes might have arisen before the divergence of maize, rice and sorghum lineages (Fig 3B).

The putative subcellular location of the plant GPDH proteins are basically assigned dependent on the existence of transmembrane domains and targeting signal [15]. In this study, we investigated the *in vivo* subcellular location of four maize GPDH proteins (ZmGPDH1, 3, 4&5) by the transient expression of GFP-tagged ZmGPDHs in mesophyll protoplasts of rice leaf. The merged image of ZmGPDH-expressed mesophyll protoplasts stained with a cytosol marker or ER-marker indicated that ZmGPDH1 and ZmGPDH3 proteins were simultaneously targeted to cytosol and ER (Fig 6). On the other hand, we couldn’t find any transmembrane regions in the amino acid sequence of ZmGPDH1 or ZmGPDH3 protein, which meant the two proteins were soluble, and since the biosynthesis of glycerolipids mainly occurred in ER, so it was conceivable that ZmGPDH1 or ZmGPDH3 might be recruited to the surface of ER for their proper functionality. To our knowledge, ZmGPDH1 and ZmGPDH3 are the first plant GPDH isoforms known to be targeted to cytosol and mainly dispersed around the ER. The earlier identified two cytosolic *Arabidopsis* AtGPDHc proteins are predicted to accumulate in the cytosol due to the absence of obvious subcellular targeting sequences and transmembrane regions, but the experimental evidence has been lacking [15,7]. The localization of ZmGPDH4 and ZmGPDH5 was clearly observed in chloroplast, which was consistent with the plasditic AtGPDHp1 and AtGPDHp2 (GLY1) proteins from *A*. *thaliana* [14,7]. Additionally, both of these genes also contain a chloroplast-targeting peptide as revealed in Fig 1.

The transcript accumulation of *ZmGPDH1-6* in various tissues of maize provided a basic understanding for their biological roles in maize development. Our study demonstrated differences in the transcriptional levels of *GPDH* genes in different organs. Among the six *GPDH* genes, *ZmGPDH1* was the most vigorous gene and expressed in all the examined tissues, which was consistent with the results of *AtGPDHc1* and both of them belonged to the cluster I in phylogenetic tree (Fig 2). The cytosolic *AtGPDHc1* gene is expressed at all developmental stages of *Arabidopsis*, and the transcripts of *AtGPDHc1* are much higher than other *AtGPDHs* in most tested tissues [15]. Northern analysis indicates that the expression of *AtGPDHp1* encoding chloroplast-targeted GPDH is induced throughout plant development, with flower tissues containing the highest level of transcripts [14]. Likewise, the plastidic *ZmGPDH4&5* genes were found active in pollen and anther. The transcript accumulation of *ZmGPDH4&5* is most likely a reflection of the G3P adjustment during plant reproductive growth stages, since pronounced lipid provision are correlated with the synthesis of anther cuticle and pollen exine [3].

Furthermore, it has been reported that GPDH enzymes play a pivotal role for lipid synthesis during the seed development in plant [3,20,41]. In this study, six *ZmGPDH* genes possessed comparatively high transcription levels at the early seed developmental stages and peaked at 10 DAF. Similar results have been reported earlier in *Arabidopsis* and cuphea: a high-level transcription of *AtGPDHp1* was detected in developing siliques [14], and the cuphea *GPDH* gene also exhibited high expression during seed development [14,42]. Additionally, it was demonstrated that G3P levels increased in young seeds and gradually declined during the late stages of seed development, when the accumulation of oil was peaked [3,43]. Hence, it is reasonable to speculate that the transcriptional patterns of *ZmGPDHs* in developing seeds are closely correlated to the variable requirement for G3P, and the high lipid levels in mature seeds may inhibit the expression of GPDHs. The above results suggest that *ZmGPDHs* are participated in the total glycerolipid biosynthesis during the maize seed development by contributing to the accumulation of G3P.

In addition to regulating plant growth, GPDHs are also important for stress adaptations, including salt, drought, and pathogen infection [7,15,18]. The promoter sequences of the maize GPDHs contained a number of *cis*-elements that likely participated in stress and defense responsiveness, such as LTR, TC-rich repeats and MBS (S2 Fig). By quantitative real-time RT-PCR, we further monitored the differential transcriptional responses of *ZmGPDHs* to abiotic stresses. Besides, the subcellular localization of ZmGPDH proteins seemed have profound effects on their stress responsiveness, as reflected by the higher level of transcripts of cytosolic *ZmGPDH1*, *2*&*3* compared to plastidic *ZmGPDH4*&*5* under stress conditions. *AtGPDHc1* have been reported to function as a stress responsive gene against salinity, dehydration and flooding, nonetheless there is no obvious change in the transcriptional level of the plastidic *AtGPDHp1* [15,14]. Meanwhile, it is worthwhile to note that *ZmGPDH6*, whose *Arabidopsis* orthologs is *AtGPDHm1* (*FAD-GPDH*), was an important member of the *ZmGPDHs* family due to its special protein structure. Shen et al. evidenced that *AtGPDHm1* was involved in abiotic stress response, and our data also showed that the transcripts of *ZmGPDH6* was highly induced when maize seedlings were subjected to stress conditions [16]. Together, our findings suggested that *ZmGPDHs* might play different roles in the abiotic stress responses in maize.

## Conclusions

In this study, six *GPDH* genes (*ZmGPDH1-6*) were identified in maize genome, among which *ZmGPDH1-5* coded for NAD^+^-dependent GPDs, whereas *ZmGPDH6* coded for FAD-dependent GPD. The diverse subcellular localization of maize GPDHs implied the potential functional distinction of maize GPDHs. The tissue specific transcript accumulation of *ZmGPDHs* demonstrated that *ZmGPDHs* were induced at the early seed developmental stages, indicating that GPDHs enzyme might be the primary contributor of G3P for oil biosynthesis in developing seeds. In addition, the transcriptional profiles of *ZmGPDHs* observed under salt, alkali, low temperature and drought revealed differential involvement of *GPDH* genes in adaptation to abiotic stresses. This result may provide important information for further studying on the biological roles of *GPDH* genes in plant development and abiotic stresses response.

## Supporting information

S1 Fig Subcellular localization of ZmGPDH1-GFP or ZmGPDH3-GFP fusion proteins. (TIF)

S2 Fig *Cis*-elements in the promoter regions of the ZmGPDHs. (TIF)

S3 Fig Multiple alignment of protein sequences of maize and *Arabidopsis* GPDHs. (TIF)

S1 Table The gene ID and chromosomal location of *GPDH* genes used in this study. (DOC)

S2 Table The syntenic relationships among maize, rice, soybean, and sorghum GPDH genes. (DOC)

S3 Table The primers used in this study. (DOC)

S1 File The predicted 3D models of ZmGPDH1-6. (ZIP)

S2 File The intron and exon boundary of *ZmGPDH1*, *ZmGPDH3*, and *ZmGPDH4&5*. (DOC)
