# Supplementary figures and images for: Glycerol-3-phosphate dehydrogenase (GPDH) gene family in Zea mays L.: Identification, subcellular localization, and transcriptional responses to abiotic stresses
Source: PLoS One. 2018 Jul 10;13(7):e0200357. doi: 10.1371/journal.pone.0200357 (PMC6039019; doi:10.1371/journal.pone.0200357)

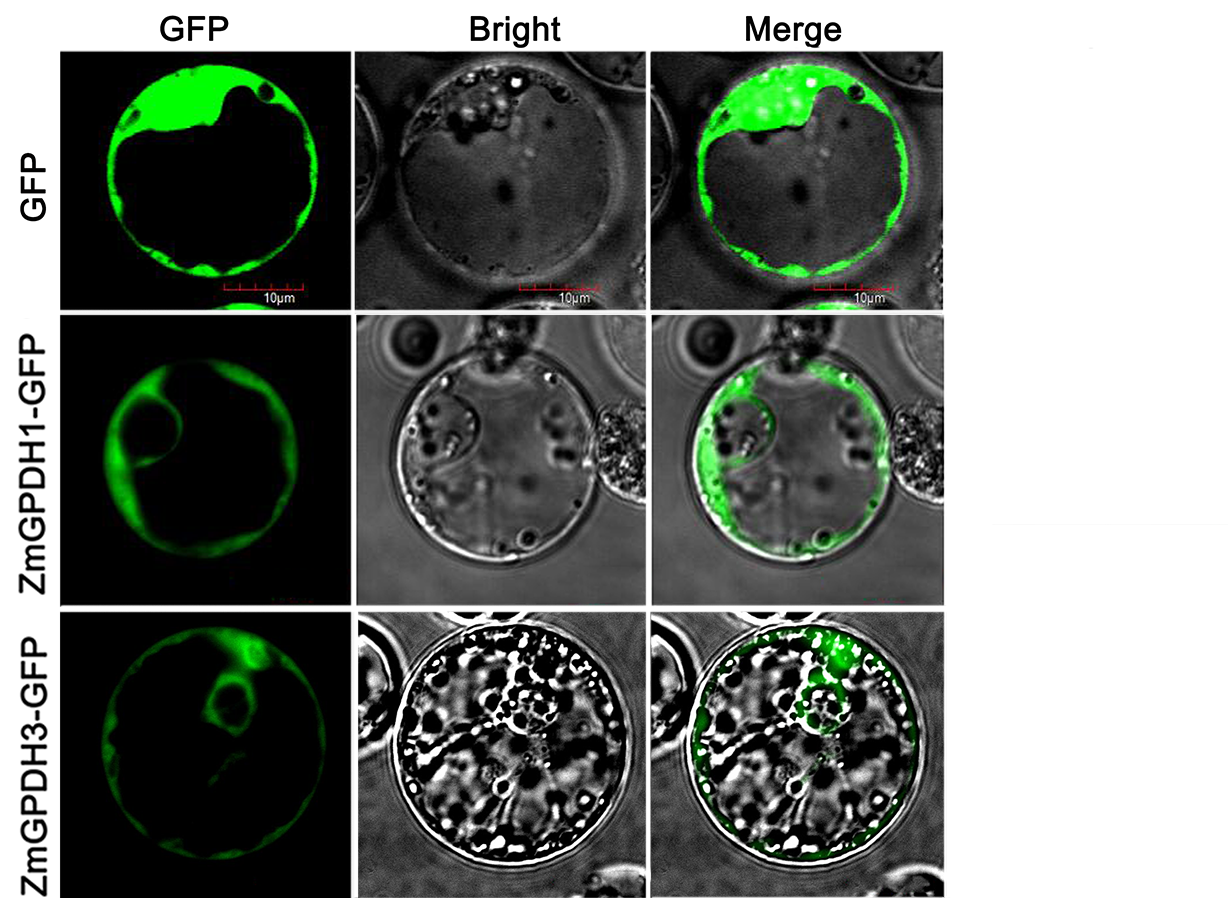

Supplement: S1 Fig — (TIF) [file pone.0200357.s001.tif]

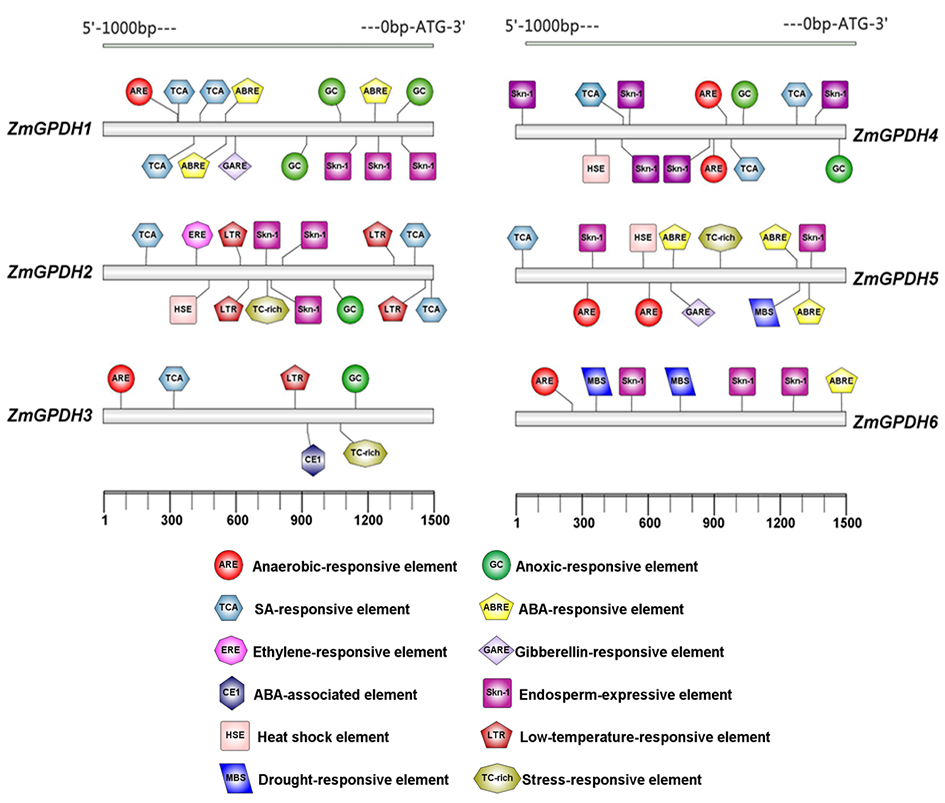

Supplement: S2 Fig — (TIF) [file pone.0200357.s002.tif]
