# Supplementary material for: Glycerol-3-phosphate dehydrogenase (GPDH) gene family in Zea mays L.: Identification, subcellular localization, and transcriptional responses to abiotic stresses
Source: PLoS One. 2018 Jul 10;13(7):e0200357. doi: 10.1371/journal.pone.0200357 (PMC6039019; doi:10.1371/journal.pone.0200357)
Supplement: S1 Table — (DOC) [file pone.0200357.s004.doc]

S1 Table. The gene ID and chromosomal location of *GPDH* genes used in this study.

| **Gene Name** | **Gene ID** | **Gene Location** | | |
| --- | --- | --- | --- | --- |
|  |  | **Chromosome** | **Start** | **End** |
| ZmGPDH1 | GRMZM2G155348_T01 | ZM3 | 152532919 | 152538365 |
| ZmGPDH2 | GRMZM2G090747_T01 | ZM8 | 171085827 | 171089416 |
| ZmGPDH3 | GRMZM2G173195_T01 | ZM8 | 120280265 | 120283927 |
| ZmGPDH4 | GRMZM6G161711_T01 | ZM3 | 144431555 | 144433915 |
| ZmGPDH5 | GRMZM2G063258_T05 | ZM7 | 21283001 | 21283001 |
| ZmGPDH6 | GRMZM2G446108_T01 | ZM10 | 96906812 | 96924949 |
| SbGPDH1 | Sobic.003G420800 | SB3 | 72591797 | 72596165 |
| SbGPDH2 | Sobic.003G326800 | SB3 | 65260392 | 65264499 |
| SbGPDH3 | Sobic.009G183300 | SB9 | 53672033 | 53675944 |
| SbGPDH4 | Sobic.002G081500 | SB2 | 8656975 | 8662094 |
| SbGPDH5 | Sobic.003G443400 | SB3 | 74139640 | 74144074 |
| SbGPDH6 | Sobic.006G029700 | SB6 | 6226081 | 6249707 |
| OsGPDH1 | LOC_Os01g71280.1 | OS1 | 41245570 | 41249927 |
| OsGPDH2 | LOC_Os01g58740.2 | OS1 | 33952187 | 33956129 |
| OsGPDH3 | LOC_Os05g41590 | OS5 | 24357908 | 24361190 |
| OsGPDH4 | LOC_Os01g74000 | OS1 | 42860708 | 42866652 |
| OsGPDH5 | LOC_Os07g12640.1 | OS7 | 7229011 | 7232294 |
| OsGPDH6 | LOC_Os04g14790 | OS4 | 8307223 | 8314255 |
| AtGPDHc1 | AT2G41540.1 | at2 | 17326170 | 17328902 |
| AtGPDHc2 | AT3G07690.1 | at3 | 2457108 | 2459552 |
| AtGPDHp1 | AT5G40610.1 | at5 | 16264861 | 16267354 |
| AtGPDHp2 | AT2G40690.1 | at2 | 16973992 | 16976458 |
| AtGPDHm1 | AT3G10370.1 | at3 | 3216368 | 3219270 |
| GmGPDH1 | Glyma.02G186600 | Gm2 | 34024695 | 34031914 |
| GmGPDH2 | Glyma.10G107100 | Gm10 | 24311327 | 24317473 |
| GmGPDH3 | Glyma.11G148900 | Gm11 | 11489777 | 11495616 |
| GmGPDH4 | Glyma.19G136100 | Gm19 | 39734235 | 39738950 |
| GmGPDH5 | Glyma.12G011200 | Gm12 | 822453 | 826257 |
| GmGPDH6 | Glyma.19G053500 | Gm19 | 8728575 | 8733034 |
| GmGPDH7 | Glyma.05G114400 | Gm5 | 30348307 | 30351975 |
| GmGPDH8 | Glyma.02G218700 | Gm2 | 40667610 | 40671395 |
| GmGPDH9 | Glyma.03G133800.1 | Gm3 | 34904861 | 34908456 |
| GmGPDH10 | Glyma.19G079400 | Gm19 | 28741811 | 28745634 |
| GmGPDH11 | Glyma.20G183100 | Gm20 | 42112199 | 42123209 |
| GmGPDH12 | Glyma.02G165500 | Gm2 | 24443552 | 24452278 |
| GmGPDH13 | Glyma.10G207700 | Gm2 | 43937582 | 43943557 |
|  | | | | |
