# Supplementary material for: Glycerol-3-phosphate dehydrogenase (GPDH) gene family in Zea mays L.: Identification, subcellular localization, and transcriptional responses to abiotic stresses
Source: PLoS One. 2018 Jul 10;13(7):e0200357. doi: 10.1371/journal.pone.0200357 (PMC6039019; doi:10.1371/journal.pone.0200357)
Supplement: S2 Table — (DOC) [file pone.0200357.s005.doc]

| **S2 Table.** The syntenic relationships among maize, rice, soybean, and sorghum *GPDH* genes | | | | | | | | | | |
| --- | --- | --- | --- | --- | --- | --- | --- | --- | --- | --- |
| **Block** | **Rice gene location** | | | **Rice Gene ID** | **Rice Gene name** | **Maize gene location** | | | **Maize Gene ID** | **Maize Gene name** |
|  | **Chr** | **start** | **end** |  |  | **Chr** | **start** | **end** |  |  |
| 8 | Os1 | 41245570 | 41249927 | LOC_Os01g71280 | *OsGPDH1* | ZM3 | 152532919 | 152538365 | GRMZM2G155348 | *ZmGPDH1* |
| 41 | Os1 | 33952187 | 33956129 | LOC_Os01g58740 | *OsGPDH2* | ZM8 | 171085827 | 171089416 | GRMZM2G090747 | *ZmGPDH2* |
| 54 | Os1 | 33952187 | 33956129 | LOC_Os01g58740 | *OsGPDH2* | ZM8 | 120280265 | 120283927 | GRMZM2G173195 | *ZmGPDH3* |
| 342 | Os5 | 24357908 | 24361190 | LOC_Os05g41590 | *OsGPDH3* | ZM8 | 171085827 | 171089416 | GRMZM2G090747 | *ZmGPDH2* |
| 341 | Os5 | 24357908 | 24361190 | LOC_Os05g41590 | *OsGPDH3* | ZM8 | 120280265 | 120283927 | GRMZM2G173195 | *ZmGPDH3* |
| 424 | Os7 | 7229011 | 7232294 | LOC_Os07g12640 | *OsGPDH5* | ZM8 | 120280265 | 120283927 | GRMZM2G063258 | *ZmGPDH3* |
| **Block** | **Rice gene location** | | | **Rice Gene ID** | **Rice Gene name** | **Rice gene location** | | | **Rice Gene ID** | **Rice Gene name** |
|  | **Chr** | **start** | **end** |  |  | **Chr** | **start** | **end** |  |  |
| 40 | Os1 | 33952187 | 33956129 | LOC_Os01g58740 | *OsGPDH2* | Os5 | 24357908 | 24361190 | LOC_Os05g41590 | *OsGPDH3* |
| **Block** | **Sorghum gene location** | | | **Sorghum Gene ID** | **Sorghum Gene name** | **Maize gene location** | | | **Maize Gene ID** | **Maize Gene name** |
|  | **Chr** | **start** | **end** |  |  | **Chr** | **start** | **end** |  |  |
| 118 | Sb3 | 72591797 | 72596165 | Sobic.003G420800 | *SbGPDH1* | ZM3 | 152532919 | 152538365 | GRMZM2G155348 | *ZmGPDH1* |
| 150 | Sb3 | 65260392 | 65264499 | Sobic.003G326800 | *SbGPDH2* | ZM8 | 171085827 | 171089416 | GRMZM2G090747 | *ZmGPDH2* |
| 402 | Sb9 | 53672033 | 53675944 | Sobic.009G183300 | *SbGPDH3* | ZM8 | 171085827 | 171089416 | GRMZM2G090747 | *ZmGPDH2* |
| 99 | Sb2 | 8656975 | 8662094 | Sobic.002G081500 | *SbGPDH4* | ZM7 | 21283001 | 21283001 | GRMZM2G063258 | *ZmGPDH5* |
| **Block** | **Sorghum gene location** | | | **Sorghum Gene ID** | **Sorghum Gene name** | **Sorghum gene location** | | | **Sorghum Gene ID** | **Sorghum Gene name** |
|  | **Chr** | **start** | **end** |  |  | **Chr** | **start** | **end** |  |  |
| 132 | Sb3 | 65260392 | 65264499 | Sobic.003G326800 | *SbGPDH2* | Sb9 | 53672033 | 53675944 | Sobic.009G183300 | *SbGPDH3* |
| **Block** | **Maize gene location** | | | **Maize Gene ID** | **Maize Gene name** | **Maize gene location** | | | **Maize Gene ID** | **Maize Gene name** |
|  | **Chr** | **start** | **end** |  |  | **Chr** | **start** | **end** |  |  |
| 385 | ZM8 | 171085827 | 171089416 | GRMZM2G090747 | *ZmGPDH2* | ZM8 | 120280265 | 120283927 | GRMZM2G173195 | *ZmGPDH3* |
| **Block** | **Rice gene location** | | | **Rice Gene ID** | **Rice Gene name** | **Sorghum gene location** | | | **Sorghum Gene ID** | **Sorghum Gene name** |
|  | **Chr** | **start** | **end** |  |  | **Chr** | **start** | **end** |  |  |
| 1 | OS1 | 41245570 | 41249927 | LOC_Os01g71280 | *OsGPDH1* | SB3 | 72591797 | 72596165 | Sobic.003G420800 | *SbGPDH1* |
| 17 | OS1 | 33952187 | 33956129 | LOC_Os01g58740 | *OsGPDH2* | SB9 | 53672033 | 53675944 | Sobic.009G183300 | *SbGPDH3* |
| 1 | OS1 | 33952187 | 33956129 | LOC_Os01g58740 | *OsGPDH2* | SB3 | 65260392 | 65264499 | Sobic.003G326800 | *SbGPDH2* |
| 148 | OS5 | 24357908 | 24361190 | LOC_Os05g41590 | *OsGPDH3* | SB9 | 53672033 | 53675944 | Sobic.009G183300 | *SbGPDH3* |
| 140 | OS5 | 24357908 | 24361190 | LOC_Os05g41590 | *OsGPDH3* | SB3 | 65260392 | 65264499 | Sobic.003G326800 | *SbGPDH2* |
| 1 | OS1 | 42860708 | 42866652 | LOC_Os01g74000 | *OsGPDH4* | SB3 | 74139640 | 74144074 | Sobic.003G443400 | *SbGPDH5* |
| 175 | OS7 | 7229011 | 7232294 | LOC_Os07g12640 | *OsGPDH5* | SB2 | 8656975 | 8662094 | Sobic.002G081500 | *SbGPDH4* |
| **Block** | **Soybean gene location** | | | **Soybean Gene ID** | **Soybean Gene name** | **Sorghum gene location** | | | **Sorghum Gene ID** | **Sorghum Gene name** |
|  | **Chr** | **start** | **end** |  |  | **Chr** | **start** | **end** |  |  |
| 372 | Gm11 | 11489777 | 11495616 | Glyma.11G148900 | *GmGPDH3* | SB3 | 72591797 | 72596165 | Sobic.003G420800 | *SbGPDH1* |
| **Block** | **Soybean gene location** | | | **Soybean Gene ID** | **Soybean Gene name** | **Soybean gene location** | | | **Soybean Gene ID** | **Soybean Gene name** |
|  | **Chr** | **start** | **end** |  |  | **end** | **start** | **end** |  |  |
| 214 | Gm2 | 34024695 | 34031914 | Glyma.02G186600 | *GmGPDH1* | Gm10 | 24311327 | 24317473 | Glyma.10G107100 | *GmGPDH2* |
| 143 | Gm2 | 34024695 | 34031914 | Glyma.02G186600 | *GmGPDH1* | Gm3 | 34904861 | 34908456 | Glyma.03G133800 | *GmGPDH9* |
| 303 | Gm2 | 34024695 | 34031914 | Glyma.02G186600 | *GmGPDH1* | Gm19 | 39734235 | 39738950 | Glyma.19G136100 | *GmGPDH4* |
| 1138 | Gm10 | 24311327 | 24317473 | Glyma.10G107100 | *GmGPDH2* | Gm19 | 39734235 | 39738950 | Glyma.19G136100 | *GmGPDH4* |
| 1297 | Gm12 | 822453 | 826257 | Glyma.12G011200 | *GmGPDH5* | Gm19 | 39734235 | 39738950 | Glyma.19G136100 | *GmGPDH4* |
| 1488 | Gm19 | 8728575 | 8733034 | Glyma.19G053500 | *GmGPDH6* | Gm19 | 28741811 | 28745634 | Glyma.19G079400 | *GmGPDH10* |
| 648 | Gm5 | 30348307 | 30351975 | Glyma.05G114400 | *GmGPDH7* | Gm19 | 8728575 | 8733034 | Glyma.19G053500 | *GmGPDH6* |
| 356 | Gm3 | 34904861 | 34908456 | Glyma.03G133800 | *GmGPDH9* | Gm10 | 24311327 | 24317473 | Glyma.10G107100 | *GmGPDH2* |
| 409 | Gm3 | 34904861 | 34908456 | Glyma.03G133800 | *GmGPDH9* | Gm19 | 39734235 | 39738950 | Glyma.19G136100 | *GmGPDH4* |
| 375 | Gm3 | 34904861 | 34908456 | Glyma.03G133800 | *GmGPDH9* | Gm12 | 822453 | 826257 | Glyma.12G011200 | *GmGPDH5* |
| 1157 | Gm2 | 43937582 | 43943557 | Glyma.10G207700 | *GmGPDH13* | Gm20 | 42112199 | 42123209 | Glyma.20G183100 | *GmGPDH11* |
