# Supplementary material for: Glycerol-3-phosphate dehydrogenase (GPDH) gene family in Zea mays L.: Identification, subcellular localization, and transcriptional responses to abiotic stresses
Source: PLoS One. 2018 Jul 10;13(7):e0200357. doi: 10.1371/journal.pone.0200357 (PMC6039019; doi:10.1371/journal.pone.0200357)
Supplement: S3 Table — (DOC) [file pone.0200357.s006.doc]

**S3 Table.** The primers used in this study.

| **Primer Names** | **Sequence (5'-3')** |  | **Description** |
| --- | --- | --- | --- |
| ZmGPDH1-F | GCTCTAGAATGGTTGGGAGCGTGCACGTC |  | cloning |
| ZmGPDH1-R | ACGCGTCGACTGGTTTTCCAAGGAGAGACG |  | cloning |
| ZmGPDH3-F | ACGCGTCGACATGGTGGGGAGCTACGCGAAC |  | cloning |
| ZmGPDH3-R | ACGCGTCGACATGGTGGGGAGCTACGCGAAC |  | cloning |
| ZmGPDH4-F | GCTCTAGAATGGAGATGGAGAACGGGCAC |  | cloning |
| ZmGPDH4-R | ACGCGTCGACGTAGAATGGAGTATGACCACCG |  | cloning |
| ZmGPDH5-F | GCTCTAGAATGGCCGCCGCCGCCGC |  | cloning |
| ZmGPDH5-R | ACGCGTCGACGACTTCCTCAACCTGGGGAAG |  | cloning |
| ZmGPDH1-qF | AAGGGAGAGTTGAGTCCTG |  | RT-qPCR |
| ZmGPDH1-qR | AGTATTCTGT AAAGCCTC |  | RT-qPCR |
| ZmGPDH2-qF | GCTAAAGGTGAATTGACA |  | RT-qPCR |
| ZmGPDH2-qR | AGAATCGAATCAGCAGGACG |  | RT-qPCR |
| ZmGPDH3-qF | AGAGGGAGCTTACGACCA |  | RT-qPCR |
| ZmGPDH3-qR | TCGCATCAGC TTTAGGCAG |  | RT-qPCR |
| ZmGPDH4-qF | GGAGGAACAGAAGAGTGG |  | RT-qPCR |
| ZmGPDH4-qR | CAGCTCCTGC CATCCTCGA |  | RT-qPCR |
| ZmGPDH5-qF | CAACCTTTCGCGGAATAGA |  | RT-qPCR |
| ZmGPDH5-qR | TTGACCTTGT ACTTCTGC |  | RT-qPCR |
| ZmGPDH6-qF | GGCTGAACAAGTGGCTACTA |  | RT-qPCR |
| ZmGPDH6-qR | ATTCGGGGCA ATGCTCTA |  | RT-qPCR |
| ZmGAPDH-qF | CCCTTCATCACCACGGACTAC |  | RT-qPCR |
| ZmGAPDH-qR | AACCTTCTTGGCACCACCCT |  | RT-qPCR |
| ZmACTIN-qF | ATCCAGGCTGTTCTTTCGTT |  | RT-qPCR |
| ZmACTIN-qR | CATTAGGTGGTCGGTGAGGT |  | RT-qPCR |
