# Supplementary material for: Glycerol-3-phosphate dehydrogenase (GPDH) gene family in Zea mays L.: Identification, subcellular localization, and transcriptional responses to abiotic stresses
Source: PLoS One. 2018 Jul 10;13(7):e0200357. doi: 10.1371/journal.pone.0200357 (PMC6039019; doi:10.1371/journal.pone.0200357)
Supplement: S2 File — (DOC) [file pone.0200357.s008.doc]

>ZmGPDH1 Gene Sequence

CGCTGGCGCATCGGTGTCGATCGCAGCAGTTCGCTTCATCACTCAAAAATCCATCTTCGTCAATAACCAGTTCGGGAAGCTGCGAGCGAAGCACTGAAGCAGATGCTTGTCTTGTCTTGTCCCGGCCCTCTCATCCTCCTCCCCCACTCCACTACGGCACTCCCGGCCCTCGCTCGCTTGCTCATTAATGGCGGGCACTGCATGGTTGGCGCCATCCATGCCCATTAACGCCTCCACCCACCTCCGGGCCGCCCCCGCCCCCATCTACTTTTATTCACGCCGATCGGGCACTTGCTTCCAATACAGCGCCGGCCGGGCCGTTGCGTCCGTCCATCCTCATCCCCATCCGCCACCCACCCCGCGCCCCTCCTTCTCACTCCAGTGCTGGCTGCCGGCCAGCTCCGCCCCCGGGGTCCTTGAGCTGGAGGTGGGTGGCGACGTAAAGGCGGCCGCTTGCTTTGCCACCGCCGGCGCTCGTCATCTCCTTACTTATTCATCCCTGCGGCCCTGCTACACATCGGTGCCGTGCCGCGCTTGATTCCGTCCTGTCCTGTGCTTGAATTGTAGGTGGCGGTGGATGACCAGCTCTCGGAGTTTTCTGAGGCGAGGATCGGAGGGCGTTGCGTTAGGTCCGCGGTTGGTGAGCGGCGGTCGGAGCGGTCTCGCGCGCGGCGGGGTCGTTCGATGACGCAGGAATCGCGCTCTGGGAGGGTGGGAATACCTCGCGCTCGCGCTCAAGGTGCTCGATTAATTTACTGCCTGAGGCTGAGGGGGGGCGCGCCTGTGAGCTGAACCGTTCCCGCTGCACTGACTCCTGAGCTCGGGAAATGGTTGGGAGCGTGCACGTCAATGGATCGGTCCACGGCGGCAATGGCACGGCCACGGAGGAGCGGCTGGACGAGCTGCGCCGGCTGCTCGGCAAGTCGGAGGGGGACCTTCTCAAGATCGTCAGCGTCGGCGCCGGCGCGTGGGGCAGCGTCTTCGCCGCGCTACTACAGGACGCGTACGGCCATTTCCGGGACAAGGTGCAGATAAGGATCTGGCGTCGCCCGGGCCGGACGGTGGACCGCTCCACCGCGGAGCACCTGTTCGAGGTGATCAACTCCAGGGAGGACGTGCTCAGGCGCCTCATCCGCCGCTGCGCCTACCTCAAGTACGTCGAGGCGCGGCTCGGAGACCGCACGCTGTATGCCGACGAGATACTCAAGGACGGCTTCTGCCTGAACATGATCGAGACGCCGCTCTGCCCTCTCAAGGTCGTCACTAACCTGCAGGAGGCCGTCTGGGATGCCGACATCGTCGTGAATGGGGTGCCGTCCACCGAGACGAGGGAGGTGTTTGATGAGATCAGCGAGTATTGGAAGGAGAGGATTAGTGTCCCGGTGATAATTTCCCTCGCCAAGGGAATTGAGGCCTCGTTGGATCCAATACCTCGTATCATTACACCTACTCAAATGATTAGCTCTGCAAgtaagaatctctcttgtctctgcctgcgtattttaaactcaatcaccacaagtttccttgaggaagctgaatggtattggttgcaagaaaaatattatgattcgtggcatgcatgataagctgcttgatcaaggtcatgattaagatcatgtttgaatgcactagagctaatagttatctactaaaattagctaaagatattcaaacagtctagctaatagattaactattagctacttttagcaattagctaatagttagctagctaattccactagcaattttttagccaactaacaattaactctaatgcattcaaacaccccctcaatagtagaattctcgatcagtttacaacaaatctatcatgtggaaagctcatttgtgttttgcaagatctctgacaaacgaacctgatgtctatagtccaagctaacaattccagtatgttacctgccacattgtccatttataagagacagcatgagcccatctattgcttgtctgttataatataactagatagtaatttgcacaattaatgccattagctgctgaacacagttagtttgtggatatgcattcaccaacaatccgttcgctccataatagtatcaatttccccttccatttgaagactgcactgaattccgattcatgtgtttagctgtctattccaattctctatttaccatatgttttatgtatactggaagtgaaacacaccaaagatgtaaatttatggcatgctcatcagtggcggatccaggattggatcaaaggaggggctataagttaaagcatgtttggaagagccaccagcggccattttgggggtgagtaccacgacgacgttctcgcaggcctgatgtactagcgtgctgcagcaggcctgccatggtgctgcttgcagcaggggcgaagccaggatccaaaattagggggaccaaattagattagggaggctgttagaagcattttttgcactatttacatgagaattagctaaaaaactaacatgtcctatagaattagggaagtattagacaggacattgctcccctgtccctctagcttgtagcctgcctcttgcgttgcgcacatgcgcgctgctaattgttagatgtgcattggccgtgcgcgctagctcatgactgtgtcctgtgtggtggtgtggatttgcactgctgaagcatggacgggtccagcgtggatgcgcgcgctaccaccttgcgtgctggtggcacggatggaggccaaatatatgtgttgaatgacacgatcagaaagaaggttgttgcctatcttagttaaccattatagtctgcatttatgttataatatactcttacgttccaaaatagttgtccttttagctcttgatttttatgtctatattcaaatgaatgatgatgaatctaaacacatatataaaacacatgcatcactcaaaaattatataaacctactaattctctaaaacgatgactactattttaagatagatgaaatatgtattaataggtgtcataggggcgaccggggctgcagccccggcagccccctcctagatccgcccctgatgctcatgacatggtgcttatcttcaatgccctgatggaaggaatggccttgttcttgaaatgcatctggttgaaacgtataaacagtttttttctgcttaatgcttggcctgcttatttgagatcagcagtacagactgatttttaaggaatgcattgcagCTGGAGTTCCAACTGAAAATATACTCTATCTTGGAGGACCAAACATCGCCTCGGAAATTTATAACAAAGAATATGCAAACGCTCGAATCTGTGGATCCAACAAGTGGAGGAAGCCTCTTGCTAAGTTTTTGAGGCAGCCACATTTCATTGTCTGGGACAACAGTGATCTTGTCACCCATGAGGTGATGGGTGGCCTGAAGAATGTCTATGCAATCGGTGCTGgtaatactaacttctgtctttttttaactgacaatattattgtacaataggcatcctactacttgatgttgaagaaaacataacaaacatgtaaatgtttttctttgcttaagttttagttgatcccttaatggtcgtccattgggctgtcactgactctctttgcctgaatagagtatgggtggatcggagtacactaaaatcagaagggctttttagaattttaagaaaagaaacaatcaatttggaaatccgatgtcgggtcaaatacttgctgcttactttttgaatagtttccattctaacgttgttttggtcataaaatggcaaggggcacaaataggatagtttcttctactttgttttactacattttctgtgagtaccagttttataaaaaaaacttgacctgcggggggtaagacagcccccgggtattgtattaagaataagaccttctcacacaggtcaagaaaacccccgaacccctgccccacacatacacagcggcatcgaagctcatgtgagaacgaccacgaccagggctgagccttagacctatgctttggcgtggaacagacgagaggatttttttaaccacagcctggaaattcgctcccacggggagtcgaactcaggacctgaggagtgctactcagaccacctaaccaactcagctagaggccctttcgcgagtaccagttttatcatatttctctaattctctttatgtttcagGAATGGTGGCAGCTTTAACAAACGAGAGTGCAACAAGCAAATCTGTATATTTTGCTCATTGCACGTCAGAGATGATATTCATTACTCATTTGCTGACAGAGCAACCTGAGAAACTCGCTGGTCCTCTGCTGGCTGACACGTATGTAACTCTCTTAAAAGGTCGCAATGCATGGTATGGGCAAATGCTTGCCAAGGGAGAGTTGAGTCCTGACATGGGTGATAGTATCAAGGGGAAGGGAATGATTCAGGtaatggaatgacttttttctttccattttgtcttgttttattctgagaagatgtcaaagctattatagtactacatccgttctcgaatatttgtcgcccgctagttcatttttgaactaaaccacgacaaataaaaaagaatggagtgagtatttgataaccctgacatttgcagccatccaattttgcttttttacacggttaccatgatcattttaccattccattggacaggGTATCTCTGCAGTTGGTGCATTTTTTGAGCTGCTTAGTCAACCCAGCTTAAGTGTGCAGCACCCAGAAGAAAACAAGCAGGTTGCTCCAGCTGAGCTATGCCCAATCCTGAAGAGGCTTTACAGAATACTGATAAAAAggtttgtgctatgtatcatcactactctccatatttggagagtttacagtgtgcttgaggagtgagtgccgcagtgcagacgaagtgagaagtgaccacttctcactcattcattttattggtttcattttactctcctgattagatatagacctgacttatgtgcaGGGAGCTCCCAGCAAGGGACATTCTTCAAGCCCTGAGGGACGAAACGATGAATGATCCTCGGGAAAGGATTGAGATGGCACAAAGCCATGCATTCTACCGCCCGTCTCTCCTTGGAAAACCATGATCTAGTTAGTCTTGTAGATTAGTAGAATATAAACGGACGCTGTGCTGCAGGTTTCTCCACTGTGCTTGCTGTCCTTGTGTCCGAGCTACATGGGGATGGGGCTTTGTCAGTTGTCGAATTCTTTGTGCTTCCTCGTTGCCATGGTGTACAAGAGATTACTGAACCAATAAATGTTCTAAGTGCATATTATATGAACACCCAATTTGAAAGTACCCGAGGAAGATTTCTTATGCATGGATTTGTTTGGTCCAACGTCATATGCTAGTTTGGGTGGCAACATCTATATTCTCATGATTGTAGATTATTATTCAAGGTATACTTAGGTTTTCTTACTTCAAAACAAGACTAAAGTTGCATC

>ZmGPDH3 Gene Sequence

GCAAGTTTGCGCTGGGATTTGGTATTTCTGGCCGCCCAACCCCACAAAACCCCCACGAAACCCACCCGCATTTCCCCTGTGCAGCTGTGCTGGCCAAGTCGTCTGGACTCTGGAGCCTGTCTGCCCGTCCCGGTTCAGCTTGCTCGCCTCGCTTATGAGTTCTGAGCTGTGACAAGAACTCAGGAGGTGGTTGACGGGTGAAGCGATGGTGGGGAGCTACGCGAACGGTGGCGGGGCTGCGCCGGCGGCGGTAGCGGCGGAGAAGCTCGACGAGCTGCGGCTGCTGCTGGGGAAGTCGGACGGCGACACGCTGCGGGTCGTCGGGGTGGGCGCCGGGGCGTGGGGGAGCGTGTTCTGCGCGCTTCTGCAGGACGCGTACGGCCGGCACCGCGACCGGGTGCAGGTGCGCGTGTGGCGCCGCGCCGGGCGCGCCGTAGACCGCGCCGACGCCGAGCGCCTCTTCGAGGTCATCAACTCCCGCGAGGACGTGCTGCGCCGGCTCATCCGCCGCTGCGCCTACCTCAAGTACGTCGAGGCGCGGCTCGGGGACCGCACGCTGCACGCCGACGAGATCCTCCGCGACGGCTTCTGCCTCAACATGCTCGACACCCCGCTCTGCCCGCTCAAGGTCGTCACCAACCTGCAGGAGGCCGTTTGGGACGCCGACATAGTTATAAACGGCCTGCCCTCCACGGAGACCAGGGAGGTATTCGGGGAGATCGGGAGGTACTGGAAGGAGCGGATCAGCCAGCCGCTCATAATCTCGCTGGCAAAGGGGATCGAGGCGTCGCTCGACCCTGTGCCCCGGATCATTACGCCCACGCAGATGATCAGCAATGCAAgtgagttctttgctcaagtctcgttgtgatctagcagtactatgttctcatggttgagttactagtttcgatgatctcgtacctctgtatacttgtggaagtgggcctaacaaatttatttgaagatctaaatgctgctccactggctctacatacagtacccctgtactgtccctgatggaatttttctggataatcatcatgatctgtatccgtggcccttcataatatttattgcttattttataattatctgggtggtactccctccatcccaaattacaagacatctcaacttttttggaggcaaaacattccaaatttgaccaaatttatgtaatattaaaataccaaataactatcattagattatttattaattatattttttataatatacctatttgatatcataaatctctgtactttttctatggttttggtttgactctccgataaagttggaatgtcttgtaatttatgatagagggagtagtatattatttgcctgctgttgattatatgaagagctagttaatgtccgctccctaagggtctgcccacgtgttatgtttgctccgcaatccttgattcccttatgaacttgtaaaaacactttagcagggtacattgggattattactgaacattgtttgttctggtttctacctgtcgcctattttaccgaactgatactaacaccctgctctttatctcaattctgtatactatactgttggtttgcttttgtggttgttgatcatggattcttactgcagCTGGAGTTCCCTTGGAGAACATTCTATATCTTGGAGGCCCAAACATTGCTTCTGAGATTTATAACAAAGAATATGCGAATGCTCGCATATGTGGAGCTGACAAGTGGAGGAAACATCTTGCAAATTTCTTGAGGCAACCTCATTTTATTGTATGGGACAATAGTGATCTCATTACTCATGAAGTCATGGGCGGCTTAAAAAACGTATATGCCATTGGGGCCGGTAagttatttgatccagaaaaatggaatttccatttgctctcttttcccagttcttatgagatacttggtcaatcattgcatatttgatgtttcatcaaaccaataaaaatgccactttggcatcttatgttcagtatttccttccctgtttcatgaagcatgccttttgcattgatttgatgctgcagtgattttgttgctgccaaatgcaatgttgcttatatttgtcagattgaacagaatcacactgtagctaatacagtatgcaaagctctcatgtcatagattaagaaaactcaaaaccagtgattaacaaaatggtagacctgggagagtgcccccatattctttatccacttctacctagttattctagagcctaaggctatccgcactcatacaactctaaatttgtactctaaaagaaatattctatccttgtcaacaaaattctttaccctatactacaatcctccgcagtcatattaactccatgtctcaaccctatatcaactaccttatattatatcattttttcaattctttccatacacacgctgctgtaagctcatggtgcagtggatacaattgctttcgtactgacgcagtgacgctaggtataccgtccgtccgctccttctacgccaaaatagcgtagcacacccagtcaaccactgcagcaaaatttgtacgctatatcgggtagcgcattacatccagtgcctcacggtcagcctaatatatgtaccatattgctgaacatggtcttatcgtgcaggtaTGGTGGCGGCCCTTACGAATGAGAGTGCAACCAGCAAATCAGTATACTTTTCACTTTGCACATCGGAAATGATATACATCACTCATCTTCTAGCACCAGAGCCCGAGAAACTTGCAGGACCCTTATTAGCTGATACATACGTCACACTGCTGAAAGGTCGCAATGCATGGTATGGGCAGAAGTTAGCTAAGGGAGAACTGACTCTAGAAATGGGGGACAGCATCAAGGGCAAAGGGACTATCCAGGtttgtgctgccaacccaccatttggacaatgtgaagtttgcttaaatgccaggtgccttttgcttaaacgcttaggctatatgctgattcttattcgtttctgtgatctaggGTGTTTCTGCAGTCAATGCATTTTATGAACTGCTGAGCCAGGGAAGCCTAAGCGTGATGCATCCAGAAACCAAGAAGGCTGTTGCTCCTGTTGAGCTATGCCCAATACTCAAAACACTATACAAAATTTTGATCAAGAGGttggaaacttgtactggtgcacttagactcattgagtcgctattgggctaattgtttaacttttacctgcaggGAGCTTACGACCAATTCCATCCTCCAGGCAATACGTGATGAATCAATGTATGATCCACGGGAAAGAATTGAGATGGCACAGAGGCAGTCTCTTTACCGGCCATCTCTTCTTGGCCTGCCTAAAGCTGATGCGAAGGCCTAAGATATGGTGTTTCTTCCTACCTGATGAGAGCTTGCAGGAGCCAGGAGGCGCTTACTGGTCTTCAAGATCCTTACGTGCCTCAGATGTGTGTTGTGTCATGCGTGTACACTAATACACTATAGAGTTGGGTGATGGCATATTTAGCATGCCAGGATAAACTTTATTGCCCGTGTCTGAAATACAAGCTGTTGTGATTCAAATTTATTTGTCACTTAGACTGGAGTAAAGATGTGTATGCCTTTTATTTTATGATGGCTTCAGAGTCTCAAAATTGGTTGTAAGATTACTCCCTGCGTCTCAAAATATAATTCGTTTTAGATTAATC

>ZmGPDH4 Gene Sequence

TTTTTTTGCCAACAAACAAAAACAAAGCAGCGGTGAAGGGAGGGGAGGAAAGGAAACAGCAACCACATGCGATTATTTCTTGGGGATTCCGCCCCTCCGACCTATCTAAGCGAACCATAACCACGGGCAGCAGGACATTATATTCTTCTGCCTGCCCTTCCGTTCAATTTCAATTTCACATTCCTTCTCTCCTCTCCTCCTGCTGCTACTAGGTTCGGGCCGTCCGTCATTGCTCTTGATTTGCCCGATTATTGCATCTCCTCTAGCTGTTGCCGCCGGCCGGGCGGCGGTGTGGTTGGCTTCTTGATCTGCATTATTTAATTCGGACCACTCCCCCGCCGTCCTCCCGCCCGCCTGCCGGAGATGGAGATGGAGAACGGGCACGCCAAGTACCGGGTGGCCGTCATTGGCAGCGGCAACTGGGGCAGCGTCGCCTCCCGCCTCATCGCCTCCAACACCGCCAAGCTGCCCTCCTTCTATGgtacgcgcattctctgtctccttctgtttcaaatattttcaattgctgccatttcgatcgcttatatcattctatatctaatttgcttcctttctctggatgcatacatataatcctctcctatgtaccagcatactaagattgttttgcctacatcttatcttatcttttaattttgtggggggtctcgatccatataaatttgtgggacgtctccatccatatacagtagacatgcagtcatgcaccaatcttccctctatgcatggacaattgggcatggtccatgccactatctgcatcttttgcccactaactgcataagcactactgctgtaaatgctatgactgatcaatgcagagtttgatgttaggtactccctgtattattattattacctacgtacacatctcttttaatttctttttttttctctctctctctgaagATGAAGTAAGGATGTGGGTGTTTGAGGAAATACTGCCTACAGGCAGGAAGCTATCTGAGTCCATTAACGAACAGAATGtaagaattagttcaagcacattaccttatcttgtttctagatgatgaaattatgttaaaatggcttacatatgtcatgacactagtaatgatgcaccgaataaaaaaaagactttattctcattggtttcaggAGAACTGCAAATACTTGCCAGGTATAAAGCTTGGAACGAATGTTATTGCCGACCCTGACTTGGAGAGCGCAGgtaatcaattaatcacagtgtcacccagctattctgatggtttagagggtagatgctactcgtaaacaacagttgagtatgtcatataatctctctcataaaacgcaaaagacaacgaacgagcatgcattagcaaacgatgacttgtcgttatcataaaaaaaacttacagcataaagctgcttgcatattgtcacataaattttatggaataaagcttttatttcccttccgcagTCAAAGACGCGAATATGCTGGTTTTTGTGACGCCCCATCAATTTGTGGAGGGTATATGTAAGAAGCTTGTAGGGAAGCTAACACCAGGAGCTGAGGCTATCTCCCTCATCAAGGGCATGGAGGTCAAGATGGAAGGGCCATGCATGATATCCAAGTTAATCGCGGATACACTTGGAATCAATTGCTGTGTGCTCATGGGTGCTAACATTGCAAACGAGgcaaccattccttcgacttgctctgcagagtttagtttcatgtagtcttcattcgtaagcaaaaataatagatggttacatgtatatatttttctagtgcagATTGCTGTCGAAGAGTTCAGTGAAGCAACAATTGGGTATAGGAAAGATAAGGAAGTGGCAAATCGATGGGCTAAACTTTTTACCACACCCTACTTCCTAGTTTCTGTCGtaagttcttctactctattctcataaataaatgtagcagagaacatacacgttatgtttcggaacatgaaaagcatcatcttaggaaatgctgaattcctttcataaaagaattatgtacgggtacataggtaccaatgaacgtcatgcttaacacctcatataaaacaaatagtattagaagcatagaagataatctatcagttataaaatgagcgttgaattatcgatgcatcttgtcctccctatgcaggCAGAAGATATTGAAGGAGTAGAGCTGTGTGGAACTCTGAAAAATATCGTGGCTATTGCAGCAGGtttttaatggatcaagtgtttcgtgtgtttacatccattcttttgctttaaacatattaatggctcatgtggcagaattgtcttgcttgtaggCCTTGTGGATGGCTTGGATATGGGAAACAATACAAAGGttgcaattcaagacatttcgaataaaagacatggttttgatggcaccgtgtgatcttatggcctcttttctttcattttttttttggttaggCTGCAATAATGAGGATTGGTTTGCGAGAAATGCGTGCTTTCTCTAAGCTTCTGTTCCCTTCAGTCAGAGACAACACGTTCTTCGAGAGCTGTGGTGTCGCCGACCTAATAACCACGTGCCgtacgtatcttcaccattgccaattaaccatccaacagatggatactgaaaagtgagtgctccatgacagTTGGTGGGAGGAACAGAAGAGTGGCTGAGGCCTTTGCACGAAATGGTGGCAAAAGGTttgttttgctttaaactacacgaaatggtggctgcggttgcatagtgataccatcttatattgctgatacaggtCTTTTGATGAACTGGAGGCAGAGATGTTGCGTGGCCAAAAACTCCAGGtaaagcaaatatatatgatgccatgcaatgcatagagaaatactctgctgggtgcatctgatctctttaattttacaggGAGTGTCCACAGCAAGGGAAGTCTATGAAGTGTTGACTTATCGAGGATGGCAGGAGCTGTTTCCTCTGTTATCAACAGTGCATGAGATCTGTATTGGGCAGTTGCCTCCTACATCGATAGTTGAATACAGTGAGCACACGCCAAATCTCTCCATCATCGGTGGTCATACTCCATTCTACTGAGGCTAGATACTGCTGTTTGGACTAGTGCTCCAGTATCACTTGTTATAAACTAAGAAATATGAGATATGTTGAATATCTGAATATGTTTTGCATATAAATATGATTTCAGTCATGTCACCTTATTAGGTGCA

>ZmGPDH5 Gene Sequence

GCCGAGTGGAATGAAGAAAGAGTGAGAAGCAGCACAAGGCGGGAAGCCGGGAACGGATAAGATAACAATCAACAAACAACAAGCCCGCGCGAGACTGTTCTTTCTCCATTTCCCAATGGCCGCCGCCGCCGCCGTCTTCTTTCCCTCCACCCCGAATCCTCGGCACCGCCTCGCCGCCGCCGCCCGCCGCCCTCCACCCAGCTTCACCGGCGCCACCGACGCCGTGCCGCTACCGGAGGACGAGGACTCCAGCGACGACGATGCCGACGACGACGGCGCGCCACGCAGGAGCGGGCGCAGGGACCGGCGCCGCGCGGTGCGCGTCGCGTGGGAGAAGCTAGTCCGGTGGTCCCGCTCCTGGCGCCGCCGCAACCGCAGCGACGTCCTCGAGACCACGCGCAAGGTagactcgctcgcaccctctatgtgtgggcttgggagggtcgcttcttcctttgctggatctgatcgagaggggctgcatggcgagtctttgctttttcaggtGGTGGTTCTCGGGGGCGGGTCGTTCGGGACGGCCATGGCCGCTCACGTGGCGGCCAAGAAGGCCGATCTCGAGGTGGCCATGCTGCTCAGGGACGACCACATCTGCCGGTCCATCAACAACGCACATGTCAATTGgttggtaacctctgctgcctttgttttctcgatccatgaggtagaatagcacttaaaaatcatctcgaacgaatagatgctcagattatccacgaacagggcacgttgtctgatgaccttagtgacctagtgatatcatcctgttattattatgaccaattaggcaatgccacgtttccggtttggaaaactagttttatttgctgtgtgcatttagccgttagcatacatcaatttgtgctgcatcctatttgcattcctcagaatacatgcgtcctgatgaggccatccccgaacaacttgcacttgttctctgcctgctcacggcaagtaatgtcggtttgtacatacggaaggtgctgtagggtcgcgctctataggccgatttggtactgtactttccatctttggtccacttccatatgattttattttatcatctaaatccgacttcagatgcacatcttatatgcatacccagagagtggcctcatcatgaaactgttttcggtgaatgtatctcaattgcagcttctggctttttactgcagCAAGTACTTATCAGAACACAGATTGCCAGAAAATATCGTCGCAACAACTAGTGCTGCTGATGCTTTAGCAGGAGCTGATTTCTGCTTCCATGCTGTTCCGGTTCAGgtattcacggttcttcacgaagcattatgctcgttgctgacatttagtcctcttgactctcttactttttgtggcagTTCAGTTCATCCTTTCTTGAAAGTATTTCAACACATGTTGATCCAAAGTTGCCATTCATATCACTTAGCAAAGGGCTGGAACTCAATACCCTTCGGACAATGTCTACAATCATCCCACGAGCATTGGGAAATCGCCGCCAACCGTTTGTTGTTCTGTCAGGACCATCATTTGCTGTAGAGTTAATGAACAAATTGCCTACAGgtctgttgatatcattaatattgttaacctgctgtcacttttttttcaagcttaatgttgatgctgagttatttgtacctgtattcctttgaacttcttcacatatctatatatgaaagaacaaacgatgggtttgaagacaccaaaacaaccctataacaatcagaaaattccgagaaaagaaaagaaaacgaattccaacaaactctcaataatcaagagcggaaacagagcttctgcactacttatattttttggtccagtttttgctgtatgttgtatggcaaatgcaactgtaattttagctactgttttttttcttcttcttgaaaacataacaaaaaatggcgtgagctgtatttgtaaccattggtatcagaatggtacaacaaatacaactgtattttaaaatccagctatgcaattgtgcaagctattcctctagtgtttgatgctcactagtctttgaatttctttctttcatcaagcacttggaatctctcaatttggactgaagtttattgtcattcaacttttaattgtagCAATGGTGGTGGCATCCAAAGACAACAAGTTGGCAAGTTCTGTTCAACAACTGTTAGCATCCCCAAATTTGAGGATAAGCACATCAAGgtaaaattgcctagctccatgagttcatcacagaagatacaacaagctacaactgcacgaatgcaaaaatgcatgccatgtaggctcgtatttactaaaactgagaaagaagatgaggtggaagatgcgtgaacgaaatgaaacacagatttttttgctcttcttactatttggttttttatgtatgagaaagagagaaaatttcttacagtttatattcaggggcatgacaatagtttctgaactattaagacaagggaaaagtgactgtgttactacctgaatattccagaacattcaataatttgcaactagtacgttcaacagctaagcataagaaataggtttccttgtttcaaattagttactaccacgcagttcaatactagtcttaggagcaatatcttgggaatttctgttgcttgagaaacttttgtccagctaaaactagcccaactaataggctatacataatatatcataccaacataaagaagtgtgatgaacgtttcttccactgttaactatttatgattttgtacacattaggatttaggaggtaggttagtagagattattttgatcatgacagttttgtacttcagcgacctgtgatactgagaaaacaataggcgaatattttgaatgtgacctatgatacttcagTGATGTTACAGGAGTAGAAATCGCAGGTGCCCTGAAGAACGTTCTTGCAATAGCTGCAGGTATAGTGGAAGGCATGAGTCTCGGGAACAACTGTATGGCTGCCCTTGTTGCCCAAGGCTGTTCAGAGATACGGTGGTTGGCGACGAAGATGGGAGCAAAGCCAACCACTCTTTCTGGCTTGTCTGGGTCCGGTGACATCATGCTCACATGCTTCGTCAACCTTTCGCGGAATAGAACAGTGGGACTACGCCTCGGTTCTGGTGAAAAGCTTGACAGAATCATGGGTTCTATGAATCAGGTgtgcagaattcctcccatggcatcagtttatgcacagggtgtcacctactcatctgtgtaacctgtggttagtggatttgtctacatgtttaggtTGCTGAAGGCGTATCAACTGCTGGGGCTGTCATTGCGTTGGCGCAGAAGTACAAGGTCAAAATGCCGGTATTGACAGCGGTGGCACGGATAATTGATAATGAGTTAACTCCAACTAAGGCGGTTTTGGAGTTAATGAATCTTCCCCAGGTacgttattaaatttgaagtttcgttctgactaccaaatgaactggatcactttgtttcacaagcacatgcacagccatcgggtccttgctctgttttaaatttgttgtgtttggctagtggctccacattttttaatttgtaaaatttaaacaataatcaggggcaatttgtctaagcaaccacatagtcttaaaagatggaaaacaaacttggtttctcctgaaatcgttcttcatgtgttgtcagtgtcttttactgttgtgttccagcgacaataatattttgatcatgcgaaaaggcgtccttgtttgcctggaagactgaaacgcacaagggttcctcactagtaaatcttccattgttcttgttcggattggtaatgcgtaacatcatgtaaaaacattctttgacaggtTGAGGAAGTCTGACGAGGCTAGCAACGGAGTTCTGCATTTTTCCACTTGGCTCCGAGTGTAGCTTGGGCCCATGGCCGACCGTATGAAAAATCTGCCAAAGACCGTTACTTTTTTTTGGGTAGCATTACATTATTAATGAAAAAATGATATCCAGTAACGGTGGTATTTAGCATTTCCTAAATTTCGAAACACATTTTCCTGTCCTTGTACACCGGCAAAAACTTCACGATGACGTTTCTTTTGAAAGAGGAAACTGTTCTCAAGCTCTGGCATTTAAATTGG

**Note**: The exon and intron was shown in capital and small letter, respectively, and the splicing site was highlighted in yellow. The start codon and [termination codon](http://www.baidu.com/link?url=9N7Xg6H2RX32WfbjclBH1KOBBYYc1vM36Xd93tiqI970hFvhBriIM_vN3i1t-xvCBRXSCm8SLG44aGTVVYdqZ_)of the open reading frame were highlighted in green.
